# Multiple Criteria Decision Analysis (MCDA) for evaluating cancer treatments in hospital-based health technology assessment: The Paraconsistent Value Framework

**DOI:** 10.1371/journal.pone.0268584

**Published:** 2022-05-25

**Authors:** Alessandro Gonçalves Campolina, Maria Del Pilar Estevez-Diz, Jair Minoro Abe, Patrícia Coelho de Soárez

**Affiliations:** 1 Departamento de Medicina Preventiva, Faculdade de Medicina FMUSP, Universidade de Sao Paulo, Sao Paulo, SP, Brasil; 2 Centro de Investigação Translacional em Oncologia, Instituto do Cancer do Estado de Sao Paulo, Faculdade de Medicina FMUSP, Universidade de Sao Paulo, Sao Paulo, SP, Brasil; 3 Departamento de Radiologia e Oncologia, Instituto do Cancer do Estado de Sao Paulo, Faculdade de Medicina FMUSP, Universidade de Sao Paulo, Sao Paulo, SP, Brasil; 4 Graduate Program in Production Engineering, Paulista University, São Paulo, SP, Brasil; University Campus Bio-Medico of Rome, ITALY

## Abstract

**Background:**

In recent years, the potential of multi-criteria decision analysis (MCDA) in the health field has been discussed widely. However, most MCDA methodologies have given little attention to the aggregation of different stakeholder individual perspectives.

**Objective:**

To illustrate how a paraconsistent theory-based MCDA reusable framework, designed to aid hospital-based Health Technology Assessment (HTA), could be used to aggregate individual expert perspectives when valuing cancer treatments.

**Methods:**

An MCDA methodological process was adopted based on paraconsistent theory and following ISPOR recommended steps in conducting an MCDA study. A proof-of-concept exercise focusing on identifying and assessing the global value of first-line treatments for metastatic colorectal cancer (mCRC) was conducted to foster the development of the MCDA framework.

**Results:**

On consultation with hospital-based HTA committee members, 11 perspectives were considered in an expert panel: medical oncology, oncologic surgery, radiotherapy, palliative care, pharmacist, health economist, epidemiologist, public health expert, health media expert, pharmaceutical industry, and patient advocate. The highest weights were assigned to the criteria “overall survival” (mean 0.22), “burden of disease” (mean 0.21) and “adverse events” (mean 0.20), and the lowest weights were given to “progression-free survival” and “cost of treatment” (mean 0.18 for both). FOLFIRI and mFlox scored the highest global value score of 0.75, followed by mFOLFOX6 with a global value score of 0.71. mIFL was ranked last with a global value score of 0.62. The paraconsistent analysis (para-analysis) of 6 first-line treatments for mCRC indicated that FOLFIRI and mFlox were the appropriate options for reimbursement in the context of this study.

**Conclusion:**

The Paraconsistent Value Framework is proposed as a step beyond the current MCDA practices, in order to improve means of dealing with individual expert perspectives in hospital-based HTA of cancer treatments.

## Introduction

Healthcare organizations around the world face a scarcity of resources to fund growing demand due to the exponential increase of technological advances. At the same time, policymakers are looking for effective ways to allocate these resources in the face of patient expectations and budget constraints [1–4].

The case of oncology is illuminating in this sense. While health gains have been made in the treatment of cancer with new therapies [5], there is concern among policymakers and health-care providers that the high costs for new cancer treatments might not be justified by the small increase in health benefits they provide over less expensive drugs [6–10].

In this context, health technology assessment (HTA) is critical in providing information about treatment alternatives to policy and decision-making, considering not only the health and economic impacts of interventions, but also the social, ethical, and institutional implications of a technology [11]. This practice has been recently defined as a “multidisciplinary process that uses explicit methods to determine the value of health technology at different points in its lifecycle. The purpose is to inform decision-making in order to promote an equitable, efficient, and high-quality health system” [12].

The interest in HTA spread around the world in the late 1980s, and the methodology has been applied on a macro (central) or meso (regional, e.g., district/county, or institutional, e.g., hospital) level at various stages of the health technology development and assessment process [12, 13]. When conducted at the meso level setting, HTA is crucial given that decentralization has been at the center stage of most health systems reforms [14]. Therefore, hospital-based HTA (HB HTA) emerged to promote HTA at the hospital level, initiating a unique methodology that has the potential to improve priority setting practices and strengthen health systems sustainability [15].

However, as decision-making in hospitals is influenced by a set of factors, including clinical benefits, overall costs, business plans, and acceptability [16], the use of economic evaluation methods, particularly cost-effectiveness analysis (CEA), to assess the incremental benefit of new medical technologies provides little guidance, since it does not capture a number of important dimensions of value [17].

In recent years, the potential of multi-criteria decision analysis (MCDA) in the health field has been discussed widely. Such discussion has led to two taskforce reports from the International Society for Pharmacoeconomics and Health Outcomes (ISPOR) [18, 19] and to extensive literature review [20–24]. Defined as both an approach and a set of methods that permit the simultaneous consideration and prioritization of different factors that may conflict during the decision-making process [16, 25], MCDA has increasingly been used to support health-care decision-making in the hospital level [18, 20]. These sets of methods offer a way to extend CEA to account for a wider variety of non-health benefits while allowing flexibility in the way that society collectively would like to make trade-offs between competing goals such as efficiency and equity [26]. A fundamental principle in MCDA is that decisions between alternatives (for example, different medical treatments) should be consistent with stakeholders’ perspectives and values [18, 19].

In this sense, an important issue when considering MCDA for HB HTA arises from the characteristics of the hospital environment–hospitals are the ‘port of entry’ of new technologies in the health-care system [14] and executives face the challenge of effectively adopting innovations with the need to improve the rationality of decision-making [15]. The urge to do so in a limited timeframe may limit an appropriate benefit-risk balance. So, HB HTA might foster decision-making by involving skilled experts that inspire clinical excellence to support priority setting in the short term [15, 16, 25].

At the same time, MCDA requires the inclusion of stakeholder’s values and expertise, and this can be realized if HTA organizes expert (or stakeholder) consultation. However, it has long been recognized that priority setting is, in reality, a value-based political process that takes place in an environment of social values and diverging interests [27]. It involves “pluralistic bargaining between different lobbies, modified by shifting political judgments made in the light of changing pressures” [28]. Therefore, the “rules” of stakeholder selection might include who can participate, whether those persons have declared conflicts of interest, when, where, and how decisions will be made, and so on. In other words, the policy entrepreneur needs to navigate the political context to create the MCDA process [27].

Experts, in the presence of vested interests, are likely to agree on a fair process, but may justifiably disagree about the range and relative importance of different values in decision-making [28]. Consequently, value assessment is not merely a question of what additional benefits to consider and possibly include in the decision-making process, but, importantly, involves how to arrive at a transparent process that elicits and accounts for the perspectives of different stakeholders in a consistent way [29, 30].

"Perspectives" and "preferences" are (inter-)related terms in MCDA literature [18, 19]. Whereas “perspectives” have been used as an umbrella term to refer to experiences, attitudes, beliefs and values [31–33]; “preferences” have been defined in a more precise way as the “assessments of the relative desirability or acceptability to patients of specified alternatives” [34, 35].

A central aspect of the debate on perspectives in MCDA is the notion of the legitimacy of the decision [28, 29, 36]. As the shared goal of the deliberation is meeting population health needs while taking into account resource limitations, decisions must be based on reasons that can be accepted to be relevant to this goal by all ‘fair-minded’ stakeholders. So, the principle of “relevance” (originally termed ‘reasonableness’ condition) [28] is fundamental for the mutual basis for decision-making [30, 36]. At the same time, in order for the principle of relevance to be met, it is important that the opinions of stakeholders are synthesized, taking into account their differences in perspectives. The paraconsistent annotated evidential logic Eτ (Logic Eτ) is a non-classical logic that allows dealing with inconsistent value judgments made by experts or stakeholders in a non-trivial way [37, 38]. Experts often face imprecise, vague, conflicting data, and even a lack of data, when conducting analyses. Classical Logic and many logical systems are not feasible for dealing with inconsistencies, at least directly [37, 39]. Thus, a language that can manipulate such inconsistencies and make sensible inferences is often needed. The Logic Eτ can be used in this purpose, in order to aggregate value judgments, even if they are contradictory [40].

The aim of this study is to illustrate how a paraconsistent theory-based MCDA reusable framework, designed to aid HB HTA, could be used to aggregate individual expert perspectives when valuing cancer treatments.

## Methods

### Study setting and MCDA approach

The study was carried out at the Instituto do Câncer do Estado de São Paulo (ICESP), a public academic hospital specializing in oncology with about 10,000 new cancer patients per year, across the state of Sao Paulo, Brazil. Since 2009, an HTA committee has been operating at ICESP, integrating essential guiding principles for good practices in hospital-based HTA to inform new technologies acquisition, such as: to state goal and scope that reflect the hospital context, to use good methods and appropriate tools that enhance transferability and to involve all relevant stakeholders in the evaluation process, ensuring independence and communication [14]. The HTA committee includes 12 members–seven physicians (oncology, hematology, radiology, infectious control expert, surgery, anesthesia, internal medicine), four administrative directors (management, executive, assistance, financial), and one pharmacy service representative. The committee meets every month on a regular basis to assess new technologies that are candidates for implementation by the hospital management. A short assessment is conducted prior to the committee meeting, but sometimes expert consultation is required to investigate further the advantages (clinical benefits and safety issues), costs, and feasibility for local adoption. After the appraisal, the final recommendations are presented to the hospital executive management for approval and budget allocation.

In order to design a reusable framework to support HTA processes at ICESP, an MCDA methodological process was adopted based on the value measurement approach and the Logic Eτ [37–39, 41], following ISPOR recommended steps in conducting an MCDA study: 1. Defining the decision problem, 2. Selecting and structuring criteria, 3. Measuring performance, 4. Scoring alternatives, 5. Weighting criteria, 6. Calculating aggregate scores, 7. Dealing with uncertainty [18, 19].

A proof-of-concept exercise focusing on identifying and assessing the global value of first-line treatments for metastatic Colorectal Cancer (mCRC) was conducted to foster the development of the MCDA framework (named Paraconsistent Value Framework), by adopting the respective scope from the latest Recommendation Reports (RR) of each selected technology [42] that has been appraised by CONITEC (Comissão Nacional de Incorporação de Tecnologias no SUS), the Brazilian national organization for HTA, or has been included in ICESP clinical guidelines for mCRC treatment [43]. As part of the technology appraisal process of CONITEC, clinical and economic evidence from a variety of sources is reviewed to assess the technology’s health benefits, the technology’s relation of benefits to costs, or “value-for-money,” and technology’s budget impact [42]. The available clinical and economic evidence from the corresponding CONITEC RR was used to populate the performance of the alternative options across the respective criteria attributes, but also, additional evidence was used, according to ICESP clinical guidelines [44–49]. The scope of CONITEC RR 324 was adopted for the cases of cetuximab and panitumumab [42], whereas clinical trials were adopted for the case of Modified Flox (mFlox), Folfox (mFolfox6), Irinotecan (mIFL) and FOLFIRI [44–46].

An expert panel, composed of key stakeholders, was appointed by the ICESP HTA committee. The composition of the group’s expertise and the numbers of the different experts were decided based on the findings of a literature review of MCDA studies in oncology that was performed to support the conduction of the present study [50] (see S1 Table for details) and the structure of the past ad hoc committees responsible for the appraisals of alternative cancer treatments at ICESP. In total, 11 experts were invited for individual face-to-face interviews, based on past ad hoc committees for cancer treatments, as these small group sizes have been shown to be large enough to represent all significant perspectives [50]. Their areas of expertise were: medical oncology, oncologic surgery, radiotherapy, palliative care, pharmacist, health economist, epidemiologist public health expert, health media expert, pharmaceutical industry, and patient advocate. This panel of experts included professionals with at least ten years of experience in decision-making at a local level.

A core component in any MCDA is the identification of criteria that decision-makers consider essential in their specific contexts. In order to ensure that a wide range of potential criteria would be included to be discussed during the HTA committee meeting, findings from the supporting previous literature review on MCDA studies in oncology were informative [51]. Drawing from this review, aimed at selecting criteria and methods for the current study, a preliminary list of evaluation criteria was compiled by the research team: therapeutic impact, efficacy, effectiveness, safety profile, innovation level, socio-economic impact, severity of disease, number of potential beneficiaries, age of target group, individual health benefits, poverty reduction, cost-effectiveness, budget impact, clinical relevance, size of affected population, unmet needs, comparative safety or tolerability, comparative patient perceived health, type of preventative benefit, type of therapeutic benefit, comparative (medical) cost of intervention, comparative (other medical) cost of intervention, comparative (non-medical) cost of intervention, quality of evidence, expert consensus, incremental benefits (including ‘equity benefits’), strategic or legal factors, therapeutic target for pediatrics, public health interest, type of medical service, completeness and consistency of reporting evidence, relevance or validity of evidence, mandate or scope of health-care system, population priorities, access, opportunity costs, affordability, system capacity, appropriate use of intervention, common goal, special interests, political, historical and cultural context [51]. This list was not intended to be exhaustive, but sought to provide a starting point for committee participants to appraise criteria they consider most relevant, based on previous studies in the field of oncology.

Then, participants of the expert panel were asked to identify the criteria considered most relevant for resource allocation in this list. During individual consultations, these experts provided feedback on the comprehensiveness, usefulness and practical limitations of each criterion. After two voting rounds in two individual interviews with each participant, a final list with 10 criteria was produced. Finally, once final criteria and their definitions were stablished, the HTA Committee, in a nominal group session, sought consensus regarding retained criteria. The relevance of criteria and their measurement scales were discussed and validated with committee members, taking into account whether a value concern was not captured in the initial set of criteria; whether a particular criterion was perceived to reflect the same value concern as another criterion; whether the underlying concern of two criteria could be reflected from a single criterion; whether the criteria meaning was clear enough to improve comprehension; whether a criterion measurement was feasible and whether the assessment of the value of one criterion would be based on the knowledge of the performance of another criterion.

From this list of proposed criteria, five were included in a specific value tree offering an organized overview of the various value concerns when evaluating new cancer medicines in an HB HTA context. Ultimately, the resulting value tree was composed into four value criteria clusters relating to the technology’s therapeutic impact (TTI), the technology’s safety profile (TSP), the technology’s socio-economic impact (SI), and severity of disease (SD). These clusters were decomposed into five sub-criteria: overall survival (OS) and progression-free survival (PFS) for TTI; grade 3 and 4 adverse events (AEs) for TSP; the cost of treatment (CT) for SD and the burden of disease the technology addresses (BoD) for SD.

Although the burden of disease criterion would have identical values across the alternative treatment options given that all of them were assessed for the same indication (mCRC), it was considered a reasonable criterion to be kept in the value framework for future applications in different diseases; however, the value tree of the present study was composed with the following criteria: OS, PFS, AEs and CT. The list of attributes and their respective definitions are shown in Table 1.

**Table 1:** Criteria definitions and range of performance.

Criterion	Sub-criterion	Definition	Metric	Range of performance
**Therapeutic impact**				
	Overall Survival	Median survival time from randomization to treatment to death	Months	1–30.51 or more 2–24.77–30.50 3–20.39–24.76 4–16.85–20.38 5–0–16.84
	Progression-Free Survival	Median survival time in which patients do not show disease progression	Months	1–12.41 or more 2–9.51–12.40 3–7.51–9.50 4–6.01–7.50 5–0–6.00
**Safety profile**				
	Grade 3 or 4 AEs *	Proportion of patients experiencing grade 3 or grade 4 adverse effects	% of patients	1–0–1.64 2–1.65–7.00 3–7.01–1.16 4–11.17–23.40 5–23.41 or more
**Socio-economic impact**				
	Cost of Treatment	Treatment cost, considering direct medical costs, based on economic evaluations carried out in Brazil	US$	1–0–5133.35 2–5133.36–11266.88 3–11266.89–15370.2 4–15370.21–20486.85 5–20486.86 or more
**Disease severity**				
	burden of disease **	Degree of severity of the disease in relation to mortality and disability derived from morbidity, which can be defined based on disability-adjusted life years (DALYs) lost	DALYs lost	1–9080001 or more 2–3600001–9080000 3–2330001–3600000 4–1330001–2330000 5–0–1330000

* Adverse effects according to Common Terminology Criteria for Adverse Events (CTCAE) v5.0—Publish Date: November 27, 2017.

** According to Global Burden of Disease (GBD) 2017 DALYs estimates.

The alternative treatment options compared in the exercise included mFlox, FOLFIRI, mFOLFOX6, mIFL, cetuximab (Erbitux®) in combination with Irinotecan and panitumumab (Vectibix®) in combination with Irinotecan. Although there is published evidence for the efficacy of bevacizumab in combination with non-oxaliplatin-based chemotherapy, and regorafenib monotherapy as treatment options [52–54], these drugs were not included in the exercise, because they were not recommended by ICESP current guidelines [43]. Overall, evidence sources used to populate the preliminary model included three randomized clinical trials (RCTs) [44–46] and respective CONITEC RR [42].

Drug costs were calculated according to prices, pack sizes, and dosage as found in the CONITEC RR, and the recommended dosage and treatment duration as reported in the respective ICESP guidelines [43] and health economic evaluations conducted in Brazil [44, 49]. DALYs estimates for colon and rectum cancer were based on The Global Burden of Diseases, Injuries, and Risk Factors Study (GBD) 2017 [55].

The sources of evidence used for identifying the performance of the treatment options across the criteria attributes are shown in Table 2.

**Table 2:** Technologies, criteria, ranges of performance and sources.

Technology	Criterion	Range of Performance	Source
**mFlox**	OS	4 (16.85–20.38)	Nebuloni DR et al. 2013
	PFS	3 (7.51–9.50)	Nebuloni DR et al. 2013
	AEs	4 (11.17–23.40)	Nebuloni DR et al. 2013
	CT	1 (0–5133.35)	Nebuloni DR et al. 2013
	BoD	1 (19000000)	Murray CJL et al 2017
**mIFL**	OS	5 (0–16.84)	Leonard BS et al. 2000
	PFS	4 (6.01–7.50)	Leonard BS et al. 2000
	AEs	5 (23.41 ou mais)	Leonard BS et al. 2000
	CT	1 (0–5133.35)	CONITEC 2018
	BoD	1 (19.000.000)	Murray CJL et al 2017
**mFOLFOX6**	OS	3 (20.39–24.76)	Tournigand C et al. 2004
	PFS	3 (7.51–9.50)	Tournigand C et al. 2004
	AEs	5 (23.41 ou mais)	Tournigand C et al. 2004
	CT	1 (0–5133.35)	CONITEC 2018
	BoD	1 (19.000.000)	Murray CJL et al 2017
**FOLFIRI**	OS	3 (20.39–24.76)	Tournigand C et al. 2004
	PFS	3 (7.51–9.50)	Tournigand C et al. 2004
	AEs	4 (11.17–23.40)	Tournigand C et al. 2004
	CT	1 (0–5133.35)	CONITEC 2018
	BoD	1 (19000000)	Murray CJL et al 2017
**Panitumumab**	OS	3 (20.39–24.76)	Douillard JY et al. 2010
	PFS	4 (6.01–7.50)	Douillard JY et al. 2010
	AEs	5 (23.41 ou mais)	Douillard JY et al. 2010
	CT	4 (15370,2–20486.85)	CONITEC 2018
	BoD	1 (19000000)	Murray CJL et al 2017
**Cetuximab**	OS	4 (16.85–20.38)	Cutsem EV et al. 2009
	PFS	3 (7.51–9.50)	Cutsem EV et al. 2009
	AEs	5 (23.41 ou mais)	Cutsem EV et al. 2009
	CT	3 (11266.88–15370.2)	CONITEC 2018
	BoD	1 (19000000)	Murray CJL et al 2017

OS = overall survival; PFS = progression free survival; AEs = grade 3 and 4 adverse events; CT = cost of treatment; BoD = burden of disease.

Following the gathering of evidence on alternatives’ performance, experts’ priorities for changes between (weights) and within criteria (scores) were captured by means of individual interviews with participants of the panel of experts (ad hoc committee).

The interviewer approached the participants to see if they would like to participate in a face to face interview. The interviews were taken in a quiet place apart from any other people that could influence the results. Subsequently, the interviewer explained the research and topic of priority setting using an information sheet and asked the respondent for informed consent. Background material introducing the scope of the exercise in more detail was sent to the participants one week before the interview. On the day of the individual interviews, the model was presented to the participant and was revised cluster by cluster in real-time through a facilitated open discussion.

Weight elicitation aimed to catch expert views of what is most important in reimbursement decision-making and which criteria should contribute most to value assessment and appraisal. A two-step swing weighting 100-point weight elicitation technique was used. The first step in the swing weighting exercise was to identify and assign 100 points to the criterion with the swing (range of performance) that matters most. This was followed by pairwise comparison between this criterion and each of the others to determine the relative importance of swings in criteria, and correspondingly allocate the points between 0 and 100 [18]. Individual weights of criteria were normalized, to sum up to the weight of the criterion category that was elicited at step one. Finally, after all, interviews were concluded, weights were aggregated using the mean of experts’ weights.

Then, performance score elicitation aimed to understand variations on how health technology is assessed and appraised with regard to its outcome for each decision criterion. Visual analog scales for performance scores were used, ranging from 0 points (worst, least relevant outcome) to 100 points (best, most relevant outcome). We asked experts their opinion about outcomes’ relevance for reimbursement and/or budget allocation of each range of performance (degree of favorable experience or belief), in a context of HB HTA. We also used a second visual analog scale and requested the experts to set their confidence on their responses about relevance for each range of performance (degree of contrary experience or disbelief), using also a scale from 0 to 100 (more details in the next subsection; see also S1 File).

Participants had to evaluate different five predefined ranges of performance (reference levels) for each criterion. These five ranges of performance were determined based on HTA cancer treatment recommendations made by CONITEC from 2013 to 2018 and based on quintile stratification. Table 2 presents the maximum and minimum values that correspond to the limits of the scale from 0 to 100. As part of framework designing, criteria ranges that were encompassed within minimum (min) and maximum (max) levels were selected. Within the min-max attribute range, we defined “lower” “intermediate” and “higher” reference levels to act as benchmarks for the value scores of 0 and 100, respectively, needed for the construction of criteria value functions (interval scales). Incorporation of such reference levels intends to establish anchors for the scales and could ensure that these value scales has enough granularity to distinguish the treatments.

A typical MCDA linear additive model was constructed. It is one of the most widely used value measurements for modeling approach [18]. It is based on global values V(a) to combine particular scores and weights brought out for selected mCRC medical treatments. The global values V(a) is usually obtained by aggregating the particular value scores received for all relevant sub-criteria. The additive aggregation is formulated by the following equation as:

$$V(a)=\sum_{i=1}^{n}\omega ivi$$

Where: V(a) global value (GV) for option a; ωi relative importance (weight) of the i criterion; vi the performance value score (partial value functions) of the i criterion.

Although a value measurement approach was adopted, the notion of preference was not used according to the Keeney and Raiffa’s Decision Theory. On the other hand, the concept of perspective was used, based on the theoretical construct of the Logic Eτ, as a way of operationalizing the attribution of scores to the performance ranges of candidate technologies, allowing the paraconsistent analysis or para-analysis (more details in the next subsection).

### Paraconsistent annotated evidential logic

The main contribution of the Logic Eτ for MCDA is that it allows dealing with inconsistent value judgments made by experts or stakeholders. Therefore, a paraconsistent methodology must enable the translation of an expert’s perspective by means of degrees of favorable personal experience and degrees of contrary personal experience, allowing the manipulation of data even if inconsistent [38]. To make it possible, it is essential that in the scoring phase of the MCDA process, participants are invited to assign two values to the performance ranges being evaluated, using 2 visual analog scales: the first corresponds to their level of belief in the relevance of that range for the eventual recommendation of a technology, and the second corresponds to their level of disbelief (uncertainty) in the attribution of the previous value. For this reason, Logic Eτ is also known as evidential bi-annotated logic, as two values are required for the constitution of its value judgment database. A detailed account of the Logic Eτ is to be found elsewhere [37, 38, 56] and in (S1 File).

Once this basic principle is met, when applying a paraconsistent framework for decision-making, one should set up the level of the requirement (control level) for the decision to be made that depends on the level of safety desired for the decision as well as the responsibility it entails [40]. These requirements establish the likelihood that a relationship observed between variables is due to chance (similar to a probability of a type-1 error in statistics). In fact, most of the scientific community is not familiar with these definitions, but they would be analogous to what we commonly understand as an alpha error of 0.5 or p value = 0.95, for example. The particularities of these requirements for analyzes based on paraconsistent logic are presented elsewhere [38, 56].

The selection of experts should look for people with different backgrounds so that the assignment of values is not a result of one single perspective. Once all invited experts assign scores for ranges of performance in each criterion (using de bi-annotated procedure) and weights to all criteria considered, the final global value score is calculated as the arithmetic means of the weights assigned by the experts [18]. Then, maximization rules (MAX operator) are applied within the groups of experts (intra-groups), and minimization rules (MIN operator) are applied in the groups of experts (between groups) [40]. The MAX operator should be applied to situations in which the favorable opinion of just one of them is enough to consider the group result as satisfactory. The MIN operator should be applied to situations where the opinions of two or more experts (or surveyed items) are all determinant, and it must be mandatory that all are favorable so that the result of the analysis is considered satisfactory [38, 40]. This way of applying the rules of maximization and minimization for decision-making is known as the min/max principle or optimistic decision because it minimizes the higher degree of certainty [40].

**Fig 1:**
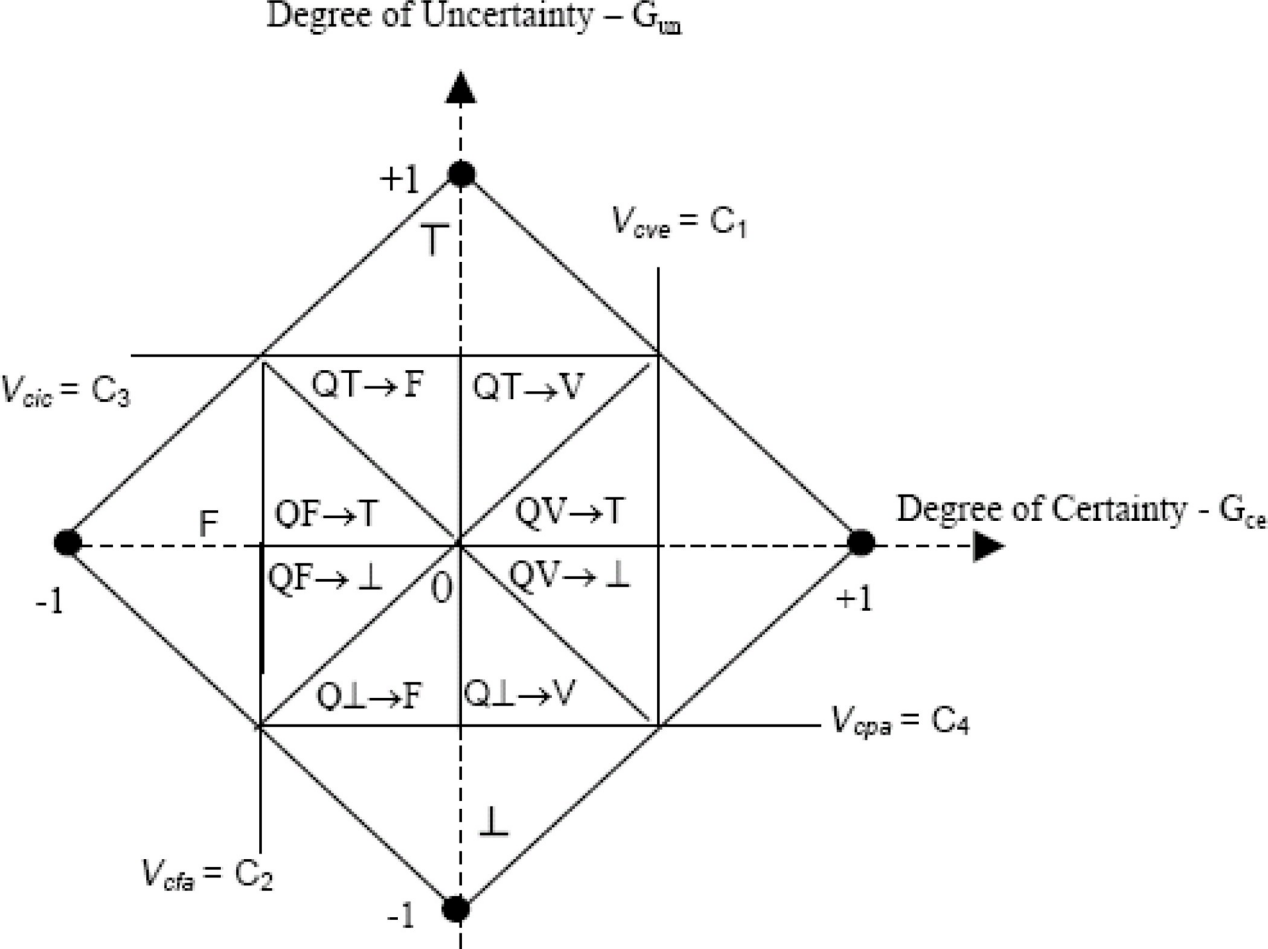
Certainty and uncertainty degrees and decision states representations in the paraconsistent lattice. V = true; F = false; T = inconsistent; ⊥ = paracomplete; QV = quasi-true; QF = quasi-false; QT = quasi-inconsistent; Q⊥ = quasi-paracomplete; C = control level; Vcic = maximum value of uncertainty control; Vcve = maximum value of certainty control; Vcpa = minimum value of uncertainty control; Vcfa = minimum value of certainty control.

The basic principle is that decision-makers might be able to identify contradictory propositions (such as particular criteria scores) in their decision problem to apply the Logic Eτ for MCDA. The contradiction in such statements can be represented with paraconsistent lattice of extreme and non-extreme states (Fig 1), with certainty and uncertainty degrees and decision states (Table 3).

**Table 3:** Extreme and non-extreme states in paraconsistent annotated evidential logic (logic Eτ).

Extreme states	Symbol	Non-extreme states	Symbol
**True**	V	Quasi-true tending to Inconsistent	QV → T
**False**	F	Quasi-true tending to Paracomplete	QV → ⊥
**Inconsistent**	T	Quasi-false tending to Inconsistent	QF → T
**Paracomplete**	⊥	Quasi-false tending to Paracomplete	QF → ⊥
		Quasi-inconsistent tending to True	QT → V
		Quasi-inconsistent tending to False	QT → F
		Quasi-paracomplete tending to True	Q⊥ → V
		Quasi-paracomplete tending to False	Q⊥ → F

V = true; F = false; T = inconsistent; ⊥ = paracomplete; QV = quasi-true; QF = quasi-false; QT = quasi-inconsistent; Q⊥ = quasi-paracomplete.

For the interpretation of the results of the para-analysis, using the lattice, first, it is necessary to identify the region in which the resultant of the analysis is found (global value) so that the decision states can be defined with their respective degrees of certainty and uncertainty associated (Fig 1). If the resultant (global value) falls into the regions of truth (V) or falsity (F), the result of the analysis is conclusive, favoring, or contradicting, respectively, the evaluated treatment or technology. If the resultant falls into the inconsistency region (T), the result of the analysis is not conclusive, indicating that the experts’ perspective is conflicting (high degree of contradiction) and that therefore new information is necessary (such as the inclusion of new criteria, for example) so that a conclusion can be reached. If the result falls into the region of para-completeness (⊥), the result of the analysis is also not conclusive, indicating that the information available is insufficient to decide and that therefore specialists with more excellent knowledge (more experience in the issues dealt with, for example) are needed so that a conclusion can be reached. Therefore, these two extreme states are indicative of the need for model revision, either in relation to the value criteria or evidence considered (state T), either in relation to the participating specialists or even in relation to the way in which they were distributed in the groups (state ⊥). Details on the lattice-based interpretation of para-analysis can be found elsewhere [37, 38, 40, 41] and in (S1 File). When the resultant fall in the other regions, inside the borders of the lattice, the resulting states are said to be non-extreme and correspond to undefined situations.

### Analysis and ethics

An MCDA tool developed in MS-Excel was used to operationalize the methodology, with spreadsheets containing all criteria, the corresponding performance categories for each criterion, and their scoring functions. The performance matrix (main evaluation sheet) of the MS-Excel tool was designed to be applicable for routine use when comparing multiple competing products in the HB HTA process. The draft MCDA tool was designed to translate the performance matrix of each product to an aggregate MCDA score according to the scoring function and weight of each criterion and indicates the uncertainty degree based on the paraconsistent analysis of each competing alternative medicines.

An explicit account of any uncertainty/limitations in the design and application of the MCDA process and the aggregate scores are interpreted and used to generate a ranking of health priorities that is intended to inform practical and rational priority setting. The uncertainty level of the MCDA value estimates was determined by means of the para-analysis, and outputs may be interpreted according to the extreme and non-extreme states (Table 3): GV falling in the true state region of the lattice is classified as pertinent (which means that the treatment is considered pertinent for reimbursement), GV falling in the false state of the lattice is classified as not pertinent (which means that the treatment is not considered pertinent for reimbursement) and all other possibilities are classified as not conclusive.

Deterministic sensitivity analysis was conducted to address parameter uncertainty by exploring the impact of different expert group arrangements and different levels of model requirements (control levels) on the pertinence of the options, and also each individual weight assigned by experts on the ranking of the options. Different scenarios were derived considering their relevance for decision-makers in the study’s context.

This study was approved by the Research Ethics Committee of University of São Paulo School of Medicine (São Paulo, Brazil). Approval number: 3.402.061. Sao Paulo, June 19, 2019. Study participants consented to participate in the study by filling out an informed consent form, approved by the aforementioned research ethics committee.

## Results

The members of the HTA committee reached consensus to distribute the variety of experts for the panel in 3 groups: clinicians (medical oncology, oncologic surgery, radiotherapy, palliative care), health-care providers (pharmacist, health economist, epidemiologist, public health expert) and society (health media expert, pharmaceutical industry and patient advocate). On consultation with these committee members, a consensus was also reached on the use of five criteria, according to researchers’ proposal and based on literature review: OS, PFS, AEs, CT, and BoD. The committee also agreed to use the swing weighting technique to assign weights to criteria and to score the performance of interventions on each criterion on a visual analog scale from 0 to 100. The committee validated the expert panel, and the individual interviews took on average 46 minutes for participants to complete the swing weighting and scoring exercise for the five criteria of the MCDA core model.

A performance matrix was elaborated, using data from the CONITEC RR and primary studies based on ICESP guidelines (Table 3). The performance of the options with the “lower” and “higher” reference levels are shown in Table 4.

**Table 4:** Performance matrix of options across criteria.

Criterion	Metric	Range (min)	Range (max)	mFlox***	mIFL****	mFOLFOX6*****	FOLFIRI	Panitumumab	Cetuximab
**Overall survival**	Median (months)	0–16.84	30.51 or more	19	14.8	20.6	21.5	23.9	19.9
**Progression free survival**	Median (months)	0–6.0	12.41 or more	7.9	7.0	8.0	8.5	7.3	8.9
**Grade 3 and 4 adverse events**	% of patients	23.41 or more	0–1.64	14.8	53.8	31	15	42	28.2
**Cost of treatment**	*US$* *	0–5133.35	81947.41 or more	3329.88	1235.13	1235.13	3545.35	16299.93	14686.03
**Burden of disease**	DALYs**	0–1330000	9080001 or more	19000000	19000000	19000000	19000000	19000000	19000000

* United States dollars (US$) using the January 2020 currency conversion rate (1 US$ = 34.02 Brazilian real (BRL)) published on the Central Bank of Brazil

** Global Burden of Disease (GBD) 2017 DALYs estimates for colon and rectum cancer [57]

***modified Flox

****modified IFL

*****modified FOLFOX6.

**Fig 2:**
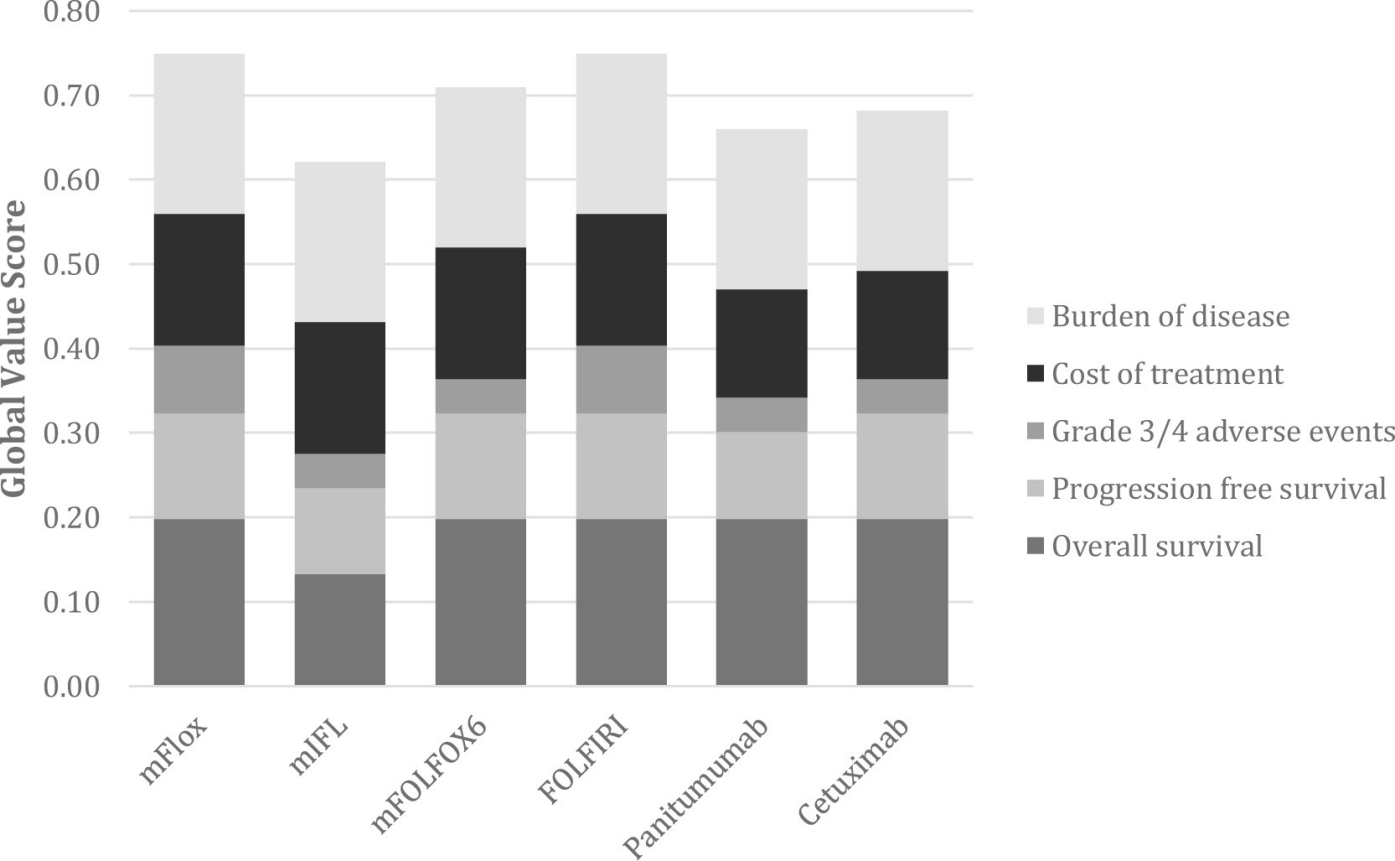
Stacked bar plot of treatments’ weighted global value scores across all criteria. mFlox = 5FU, oxaliplatin, leucovorin; mIFL = irinotecan, 5FU, leucovorin; mFOLFOX6 = 5FU, oxaliplatin, leucovorin; FOLFIRI = 5FU, oxaliplatin, irinotecan, leucovorin.

The highest weights were assigned to the criteria “OS” (mean 0.22), “BoD” (mean 0.21), and “AEs” (mean 0.20), and the lowest weights were given to “PFS” and “CT” (mean 0.18 for both). Estimates of perceived value for the six medicines appraised, resulting from the multiplication of normalized weights by scores and summation across criteria (linear additive model), ranged from 0.62 to 0.75 on a scale of 0 to 1 (Fig 2). FOLFIRI and mFlox scored the highest global value score of 0.75, followed by mFOLFOX6 with a global value score of 0.71. mIFL was ranked last with a global value score of 0.62, partially due to its worst performance on OS (Fig 2). The scores assigned to different ranges of performance and the relative weights assigned to the different criteria can be found in the (S2 and S3 Tables).

**Fig 3:**
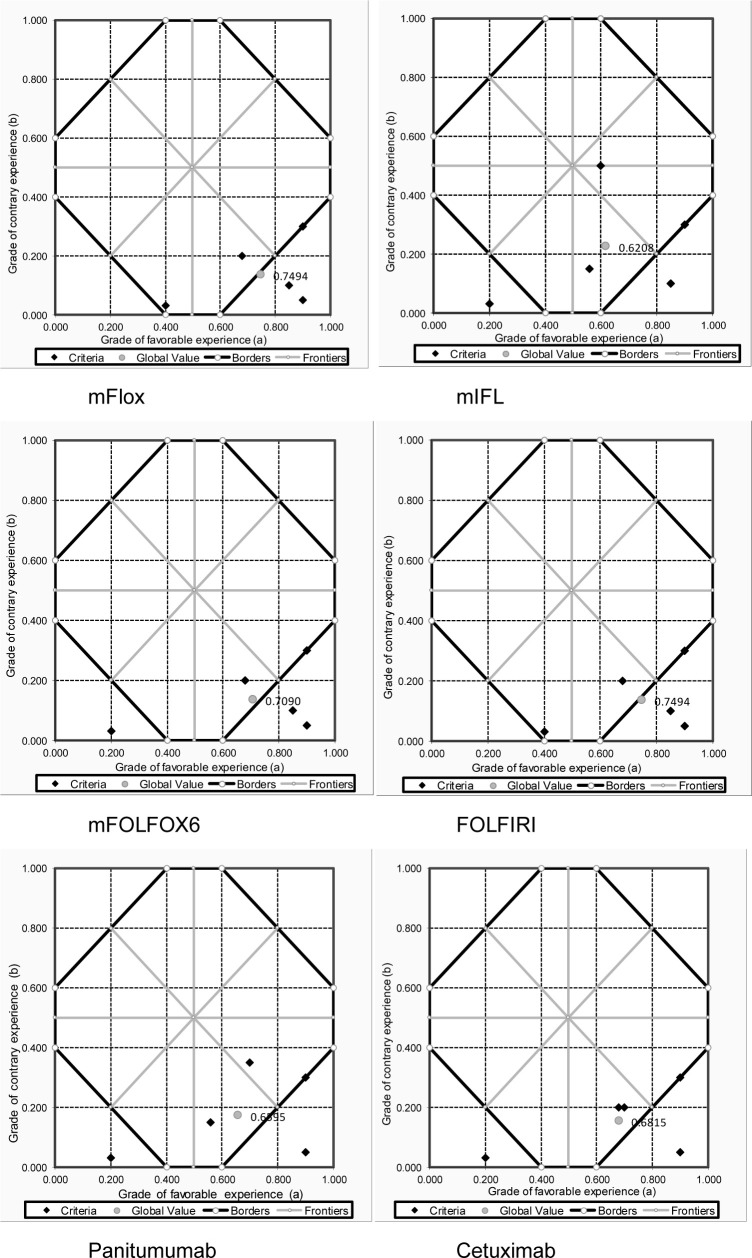
Paraconsistent analysis of first line metastatic colorectal cancer treatments. 1 = modified Flox (5FU, oxaliplatin, leucovorin); 2 = modified IFL (irinotecan, 5FU, leucovorin); 3 = modified FOLFOX6 (5FU, oxaliplatin, leucovorin); 4 = FOLFIRI (5FU, oxaliplatin, irinotecan, leucovorin); 5 = Panitumumab; 6 = Cetuximab.

Fig 3 shows the para-analysis of the six first-line treatments for mCRC. Two of these treatments (mFlox and FOLFIRI) fell into the truth region of the lattice, indicating that they are the appropriate options for incorporation and reimbursement in the context of this study. The other treatments (mIFL, mFolfox6, panitumumab, and cetuximab) fell into the lattice’s inconclusive region, indicating that one cannot conclude for or against the pertinence of these treatments, based on the decision problem addressed and the level of the requirement established for the decision (control level).

**Fig 4:**
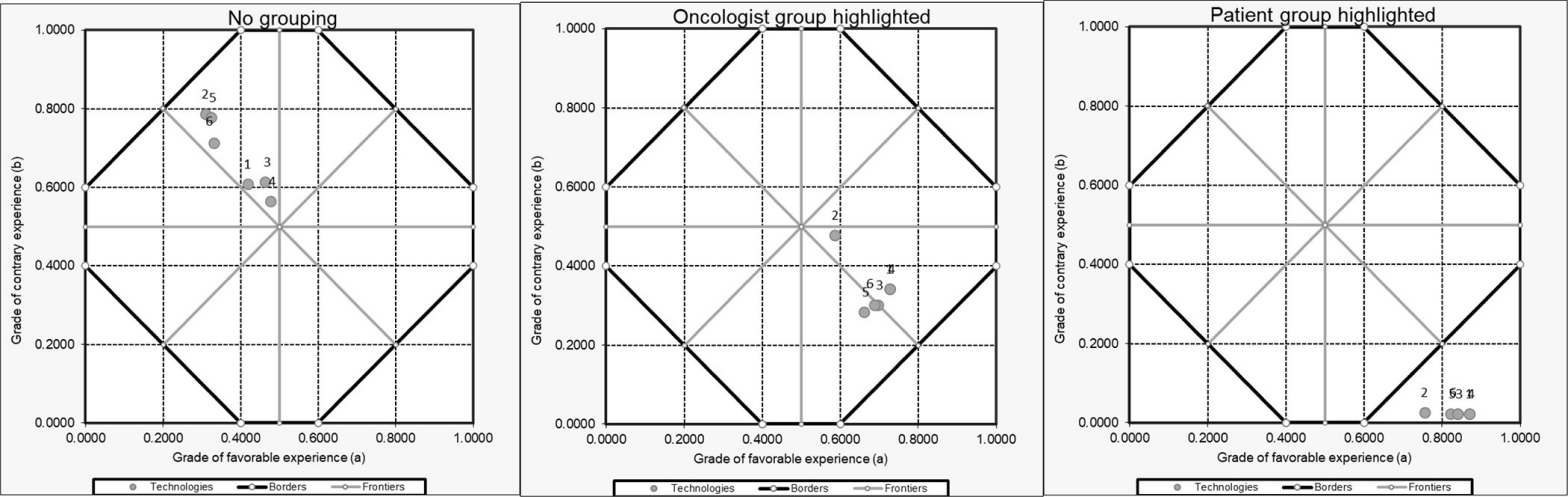
One-way sensitivity analysis on different expert group arrangements for first line metastatic colorectal cancer treatments. 1 = modified Flox (5FU, oxaliplatin, leucovorin); 2 = modified IFL (irinotecan, 5FU, leucovorin); 3 = modified FOLFOX6 (5FU, oxaliplatin, leucovorin); 4 = FOLFIRI (5FU, oxaliplatin, irinotecan, leucovorin); 5 = Panitumumab; 6 = Cetuximab.

Deterministic sensitivity analysis was conducted by exploring the impact of perspectives changes on the pertinence of the options. Fig 4 shows the result of 3 scenarios and their respective impacts. In the first, without the establishment of groups, the 11 experts were considered independently as an isolated perspective each. In the second, two groups were created, one containing only the expert in oncology and the other with all the other experts grouped. In the third, two groups were also created, but one with only the expert representing the patient’s view (patient representative) and the other with the other experts grouped. In the first scenario, the result of the analysis fell into the inconclusive region (quasi-false tending to inconsistent). In the second scenario, with the oncologist’s perspective highlighted, the result also fell into an inconclusive region, but in a state tending to the truth and favoring the pertinence of all alternatives (quasi-true tending to inconsistent and quasi-true tending to para-complete). In the third scenario, with the highlight of the patient’s perspective, the result of the analysis fell into the region of truth (true extreme state), reinforcing the pertinence of all the options considered.

Deterministic sensitivity analysis was also conducted to explore the impact of baseline weight changes on the ranking of the options (figures shown in S1 Fig). To simulate the impact of these changes, we substituted the mean weight, applied in the base case, by individual weights assigned by each of the 11 experts to explore the impact of each perspective. The simulations did not change the rank of the alternatives based on the global value of each one. On the other hand, changes in control levels, ranging from 0.50 to 0.70, led to the modification of the alternatives considered pertinent from the paraconsistent analysis (see S2 Fig). Therefore, conclusions were fairly robust as treatment rankings were not influenced by changes of perspectives on any of the baseline normalized weights, but at the same time, the pertinence was sensitive to the model’s requirement level.

## Discussion

This proof of concept study exemplifies how a paraconsistent MCDA framework can provide a means to integrate a wide range of different experts’ perspectives into the priority setting of cancer treatments in an HB HTA context. The main contribution of this work is to explore a methodology to address the aggregation of multiple perspectives in a MCDA framework designed to evaluate cancer treatments at a local level.

The use of HTA has expanded significantly over the past decades and value frameworks is now been used to support the assessment and appraisal of new medical technologies [17, 26, 32]. In general, value frameworks adopt multiple criteria approaches and MCDA process has guided the construction of these tools to support the allocation of health resources. In this paper we outlined a new value framework to focus on healthcare decision making related to cancer treatment. A specific value-based model was developed, taking the form of a value tree for the purpose of assessing the value of new medicines in the context of HB HTA.

In a previous study, a generic value framework was proposed to capture a comprehensive set of value concerns that can be adapted to different decision-making contexts, including different health conditions [17]. The different components of the resulting value tree, which was called “Advance Value Tree”, was similar to the ones of the present study: (a) burden of disease, (b) therapeutic impact, (c) safety profile, (d) innovation level, and (e) socioeconomic impact. The “Advance Value Tree”, has been developed under the auspices of the Advance-HTA Project, which applied MCDA methodological process to develop a new value framework (the “Advance Value Framework”).

The “Advance Value Framework” links the use of MCDA with opportunity costs providing a framework to inform negotiations leading to coverage and reimbursement decisions, in a non-supplementary way. The purpose is to provide an alternative approach to economic evaluation (CEA) that could be used do derive the different options’ incremental cost value ratio (ICVR) drawing from a “clean state” MCDA approach. In a different way, the Paraconsistent Value Framework, in addition to being cancer-specific, inform priority setting decisions at a local level, in order to offer a supplementary tool to HTA practices, encompassing societal perspective, while incorporating views from the wider stakeholder community, such as expert panels, following a sound methodology.

In another study, the “Advance Value Framework” was applied for evaluating a set of drugs for the treatment of metastatic colorectal cancer following first line chemotherapy [57]. Cetuximab scored the highest global value score, followed by panitumumab. Aflibercept in combination with FOLFIRI scored the lowest global value score. In terms of value-for-money, aflibercept in combination with FOLFIRI was shown to be dominated by panitumumab, both of which were shown to be dominated by cetuximab. The therapeutic impact cluster (three attributes) totaled overall a relative weight of 0.47 (OS = 28.9), the safety profile cluster (AEs) a relative weight of 0.23, the innovation level cluster a relative weight of 0.19, and the socioeconomic impact cluster (medical costs impact) a relative weight of 0.12.

Unlike that study, in the present study FOLFIRI and mFlox scored the highest global score, outperforming cetuximab and panitumumab global scores. On the other hand, similarly, the highest relative weights were assigned to criteria OS (0.22) and AEs (0.21), the lowest value was assigned to CT (0.18). Two reasons explain these differences: the variation in the value judgments of the different stakeholders included in each study and the variation in the performance of the technologies according to the references that populated the models. Furthermore, although the highest relative weights of the criteria were attributed to similar criteria (OS and AEs), and in the present study CT also received the lowest relative weight, quantitatively these values were different. In the present study, the weight of criteria related to therapeutic benefit and safety were lower, compared to the “Advance Value Framework” study, while the weight attributed to economic aspects (CT) was greater in the present study. The greater weight attributed to the economic related criterion in the present study may be associated in part to the incorporation of the purchase price and administration of the drug, differently from the “Advance Value Framework” study.

Another important difference between these studies is that the “Advance Value Framework” study provides a cost benefit plot of global values, based on Multi Attribute Value Theory [17, 57], while the current study provides an uncertainty analyses of individual value judgments, based on Logic Eτ theory [37, 38]. Although the theoretical constructs and application purposes are different (one more focused on value for money analysis and the other more focused on priority setting under uncertainty at a local level), future studies may investigate possibilities of complementary uses of the two approaches.

Sensitivity analyzes helped to explore the perspectives being considered, insofar as the variations, and even contradictions of these individual perspectives, is a central component of Logic Eτ. In the first scenario, when we did not group the participants (panel members), the importance of the perspectives in influencing the global value is considered the same. In the case of our study, this scenario led to a lack of definition, making a final analysis impossible, when comparing the 6 treatments according to Fig 4 (“no grouping”). When we put the perspective of the clinical oncologist isolated in a group (which gives more significant influence and capacity for determination to this perspective), the resultant of all technologies continues in a region of undefinition, but in sub-regions tending to the truth or favoring the pertinence of treatments (Fig 4, “oncologist highlighted”). When we isolate the perspective of the patient representative in a group (condition of most significant influence), we observe that the result of all treatments falls in the region of truth, which reveals the interest in incorporating and reimbursing all options by this expert (Fig 4, “patient highlighted”). These interpretations are possible due to the MAX and MIN rules applied in para-analysis [40].

Overall, a set of different treatment options for the indication of mCRC at first-line were assessed and ranked based on their global value scores (Fig 2). These scores acted as value indexes, comprised of the performance of the alternative treatment options against a specific set of criteria. At the same time, these metrics were adjusted for the relative pertinence according to different experts’ perspectives, as reflected by the para-analysis (Fig 3) although the participants might at first have had opposing views and beliefs in regard to their judgments, the methodology allowed to make these differences explicit and to use contradictory information to support decision-making.

Although the Paraconsistent Value Framework is not grounded on utility or value theory [58], the implementation of this MCDA methodology at a local level (HB HTA) could take place in the form of a supplementary “incremental” mode to other assessment processes [17, 26], such as cost-effectiveness analysis (CEA). The main purpose of the Paraconsistent Value Framework is to enable an easier exploration of alternatives by decision-makers in real-world, through extensive expert engagement and an encompassing capture of value perceptions and experiences in health-care.

A central strength of this methodology, as explored through this case study, is the development of the evaluation model with a group of relevant experts, which provided relevance to varying ranges of health interventions’ performance, based on their previous experience in health services. Furthermore, the framework was adjusted in that values were not restricted to the appraisal committee or evaluation board responsible for decision-making. Instead, health-care experience and scientific evidence were gathered in a way that the wider stakeholder community (HB HTA committee and decision-makers) could potentially reuse the built model in the future evaluation of cancer treatments, allowing for other voices to take part of the process.

This study should be considered in light of its limitations. First, although the methodological process we adopted is generally in alignment with recent good practice guidelines on the use of MCDA for health care decision-making [18, 19], the review of the analysis was not done in this first exercise. So, it was not possible to obtain committee members’ feedbacks and to evaluate if they were positive about its use and easy of results interpretation.

Second, the inclusion of OS and PFS in the model would entail double counting effects [19]; however, due to the importance of these two measures for decision-making in oncology, committee members insisted on the relevance of maintaining them in the model. In this regard, some of these participants argued that PFS could possibly capture concerns about quality of life.

Third, it is crucial to consider that we used un-synthesized data for performance assessment so that estimates of treatment effects should be considered with caution. Alternatively, relative treatment effects should be estimated through indirect treatment comparisons making use of indirect evidence through a common comparator or network meta-analysis.

Fourth, setting ranges of performance, based on which treatment scores were derived, was challenging. Although we recognize the importance of expert opinions, we did not want to set levels that could be perceived as extremely optimistic or pessimistic, or even regarded as “ideal” levels. So, we tried to be as objective as possible when setting these ranges, and we decided to adopt the percentile distributions of previous CONITEC HTA processes for cancer treatments. Anyway, these levels were validated by the committee members.

Fifth, the use of five ranges of performance for each criterion may not guarantee the necessary granularity for partial value functions, in order to make it capable of differentiating alternatives with very close performances. However, for HB HTA applications of the methodology, we consider that this would not compromise the use of the framework since even partial MCDA has been recommended for this type of application [59].

Sixth, it is important to state that some would argue that correct application of the swing weighting technique would involve a third step: consistency check [60, 61]. Thus, the method of eliciting the criteria weights must be seen in light of this limitation, since consistency check was not performed. Although we did not perform this step, the application sought to follow the best practice recommendations for MCDA studies [19].

Finally, it should be clear that this is a simulation exercise illustrating the application of a new cancer value framework that does not act as a panacea for challenges relating to appropriate evidence collection, combination of different expert experiences (or multi-stakeholder perspectives). Instead, the paraconsistent MCDA framework should be seen as a tool to understand, construct, and analyze perspectives in light of vested interests, contradictory information, and already existing evidence.

The Paraconsistent Value Framework proposes a comprehensive methodology to consider in making health-care choices when dealing with variations in expert opinions [62] is vital to increase the legitimacy and transparency of decisions. Moreover, the approach is also able to reveal gaps in available data and the need to align better experts (or stakeholders) with decision problem needs. Overall, the framework encouraged a better analysis of the issues that troubled panelists subconsciously [63], rendering them more explicit in this assessment.

Further research is needed to position the paraconsistent MCDA framework within a frame of reference for the value assessment of cancer treatments. Thereby future studies should field test the framework for other types of Cancer and compare treatments for different oncological diseases.

## Conclusion

The Paraconsistent Value Framework was designed to illustrate how cancer treatments could be valued from different expert perspectives in HB HTA. The framework is proposed as a step beyond the current MCDA practices, designed to aid HB HTA, in a supplementary way. Further testing and validation are needed to build up the MCDA approach combined with paraconsistent methodology in order to improve the means to deal with uncertainty in priority setting and health-care decision-making.

## Supporting information

S1 Table Search strategy for MCDA studies in oncology. (DOCX)

S2 Table Performance scores (bivalued annotations) assigned by experts. (DOCX)

S3 Table Criteria weights assigned by experts. (DOCX)

S1 Fig One-way sensitivity analysis on criteria weights for first line metastatic colorectal cancer. (DOCX)

S2 Fig One-way sensitivity analysis on control levels for first-line metastatic colorectal cancer. (DOCX)

S1 File Primer on annotated evidential logic. (DOCX)

S2 File Cancer model and paraconsistent MCDA tool. (XLSX)

## References

[1] Drummond M, Sculpher M, Torrance G, O’Brien B, Stoddart G. Methods for the economic evaluation of health care programmes. Oxford: Oxford University Press; 2005.

[2] Stephens JM, Handke B, Doshi JA. International survey of methods used in health technology assessment (HTA): does practice meet the principles proposed for good research? Comparative Effectiveness Research. 2012;2012: 29–44. doi: 10.2147/CER.S22984

[3] NICE. Guide to the processes of technology appraisal. 2018. Available: https://www.nice.org.uk/Media/Default/About/what-we-do/NICE-guidance/NICE-technology-appraisals/technology-appraisal-processes-guide-apr-2018.pdf 27905710

[4] Angelis A, Lange A, Kanavos P. Using health technology assessment to assess the value of new medicines: results of a systematic review and expert consultation across eight European countries. European Journal of Health Economics. 2018;19: 123–152. doi: 10.1007/s10198-017-0871-0 28303438 PMC5773640

[5] Schrag D. The price tag on progress—Chemotherapy for colorectal cancer. New England Journal of Medicine. Massachusetts Medical Society; 2004. pp. 317–319. doi: 10.1056/NEJMp048143 15269308

[6] Bach PB. Limits on medicare’s ability to control rising spending on cancer drugs. New England Journal of Medicine. Massachussetts Medical Society; 2009. p. 626. doi: 10.1056/NEJMhpr0807774 19176475

[7] Mariotto AB, Yabroff KR, Shao Y, Feuer EJ, Brown ML. Projections of the cost of cancer care in the United States: 2010–2020. J Natl Cancer Inst. 2011/01/12. 2011;103: 117–128. doi: 10.1093/jnci/djq495 21228314 PMC3107566

[8] Burki TK. Rising cancer drug costs in the USA. The Lancet Oncology. 2017;18: e652. doi: 10.1016/S1470-2045(17)30805-7 29066009

[9] Mariotto AB, Enewold L, Zhao J, Zeruto CA, Yabroff KR. Medical Care Costs Associated with Cancer Survivorship in the United States. Cancer Epidemiol Biomarkers Prev. 2020;29: 1304–1312. doi: 10.1158/1055-9965.EPI-19-1534 32522832 PMC9514601

[10] Butler SS, Winkfield KM, Ahn C, Song Z, Dee EC, Mahal BA, et al. Association of Rising Cost and Use of Oral Anticancer Drugs With Medicare Part D Spending From 2013 Through 2017. Am Soc Clin Oncol Educ Book. 2017;77: 18–22. doi: 10.1001/jamaoncol.2019.4720

[11] Banta D. What is technology assessment? International Journal of Technology Assessment in Health Care. Int J Technol Assess Health Care; 2009. pp. 7–9. doi: 10.1017/S0266462309090333 19519979

[12] O’Rourke B, Oortwijn W, Schuller T. The new definition of health technology assessment: A milestone in international collaboration. International Journal of Technology Assessment in Health Care. 2020; 1–4. doi: 10.1017/S0266462320000215 32398176

[13] Oortwijn W, Mathijssen J, Banta D. The role of health technology assessment on pharmaceutical reimbursement in selected middle-income countries. Health Policy. 2010;95: 174–184. doi: 10.1016/j.healthpol.2009.12.008 20074829

[14] Sampietro-Colom L, Lach K, Pasternack I, Wasserfallen JB, Cicchetti A, Marchetti M, et al. GUIDING PRINCIPLES for GOOD PRACTICES in HOSPITAL-BASED HEALTH TECHNOLOGY ASSESSMENT UNITS. International Journal of Technology Assessment in Health Care. 2016;31: 457–465. doi: 10.1017/S0266462315000732 26899230

[15] Cicchetti A, Iacopino V, Coretti S, Fiore A, Marchetti M, Sampietro-Colom L, et al. Toward A Contingency Model for Hospital-Based Health Technology Assessment: Evidence from Adhophta Project. International Journal of Technology Assessment in Health Care. 2018;34: 205–211. doi: 10.1017/S0266462318000119 29656722

[16] Tal O, Booch M, Bar-Yehuda S. Hospital staff perspectives towards health technology assessment: Data from a multidisciplinary survey. Health Research Policy and Systems. 2019;17: 72. doi: 10.1186/s12961-019-0469-3 31337398 PMC6651984

[17] Angelis A, Kanavos P. Multiple Criteria Decision Analysis (MCDA) for evaluating new medicines in Health Technology Assessment and beyond: The Advance Value Framework. Social Science and Medicine. 2017;188: 137–156. doi: 10.1016/j.socscimed.2017.06.024 28772164

[18] Thokala P, Devlin N, Marsh K, Baltussen R, Boysen M, Kalo Z, et al. Multiple criteria decision analysis for health care decision making—An introduction: Report 1 of the ISPOR MCDA Emerging Good Practices Task Force. Value in Health. 2016;19: 1–13. doi: 10.1016/j.jval.2015.12.003 26797229

[19] Marsh K, Ijzerman M, Thokala P, Baltussen R, Boysen M, Kaló Z, et al. Multiple Criteria Decision Analysis for Health Care Decision Making—Emerging Good Practices: Report 2 of the ISPOR MCDA Emerging Good Practices Task Force. Value in Health. 2016;19: 125–137. doi: 10.1016/j.jval.2015.12.016 27021745

[20] Thokala P, Duenas A. Multiple criteria decision analysis for health technology assessment. Value in Health. 2012;15: 1172–1181. doi: 10.1016/j.jval.2012.06.015 23244821

[21] Marsh K, Lanitis T, Neasham D, Orfanos P, Caro J. Assessing the value of healthcare interventions using multi-criteria decision analysis: A review of the literature. PharmacoEconomics. Adis International Ltd; 2014. pp. 345–365. doi: 10.1007/s40273-014-0135-0 24504851

[22] Adunlin G, Diaby V, Xiao H. Application of multicriteria decision analysis in health care: A systematic review and bibliometric analysis. Health Expectations. 2015;18: 1894–1905. doi: 10.1111/hex.12287 25327341 PMC4402125

[23] Vorobiev P, Holownia M, Krasnova L. Multi-Criteria Decision Analysis (MCDA) and Its Alternatives in Health Technology Assessment. Journal of Health Policy and Outcomes Research. 2015; 34–43. doi: 10.7365/jhpor.2015.1.4

[24] Oliveira MD, Mataloto I, Kanavos P. Multi-criteria decision analysis for health technology assessment: addressing methodological challenges to improve the state of the art. European Journal of Health Economics. 2019;20: 891–918. doi: 10.1007/s10198-019-01052-3 31006056 PMC6652169

[25] Martelli N, Hansen P, van den Brink H, Boudard A, Cordonnier AL, Devaux C, et al. Combining multi-criteria decision analysis and mini-health technology assessment: A funding decision-support tool for medical devices in a university hospital setting. Journal of Biomedical Informatics. 2016;59: 201–208. doi: 10.1016/j.jbi.2015.12.002 26705065

[26] Angelis A, Kanavos P. Value-Based Assessment of New Medical Technologies: Towards a Robust Methodological Framework for the Application of Multiple Criteria Decision Analysis in the Context of Health Technology Assessment. Pharmacoeconomics. 2016;34: 435–446. doi: 10.1007/s40273-015-0370-z 26739955 PMC4828475

[27] Mitton C, Donaldson C. Health care priority setting: Principles, practice and challenges. Cost Effectiveness and Resource Allocation. 2004;2: 3. doi: 10.1186/1478-7547-2-3 15104792 PMC411060

[28] Daniels N. Accountability for reasonableness. British Medical Journal. BMJ; 2000. pp. 1300–1301. doi: 10.1136/bmj.321.7272.1300 11090498 PMC1119050

[29] Daniels N, Sabin JE. Accountability for reasonableness: an update. BMJ. 2008;337: a1850. doi: 10.1136/bmj.a1850 18845595

[30] Baltussen R, Jansen MP, Mikkelsen E, Tromp N, Hontelez J, Bijlmakers L, et al. Priority setting for universal health coverage: We need evidence-informed deliberative processes, not just more evidence on cost-effectiveness. International Journal of Health Policy and Management. Kerman University of Medical Sciences; 2016. pp. 615–618. doi: 10.15171/ijhpm.2016.83 27801355 PMC5088720

[31] Janssens R, Overbeeke E van, Verswijvel L, Meeusen L, Coenegrachts C, Pauwels K, et al. Patient involvement in the lifecycle of medicines according to Belgian stakeholders: The gap between theory and practice. Frontiers in Medicine. 2018;5: 285. doi: 10.3389/fmed.2018.00285 30364285 PMC6193089

[32] Campolina AG. Value-based medicine in oncology: the importance of perspective in the emerging value frameworks. Clinics. 2018;73: e470s. Available: doi: 10.6061/clinics/2018/e470s 30540119 PMC6256994

[33] Wiffen P. Value or cost: Looking for the wider perspective. European Journal of Hospital Pharmacy. BMJ Publishing Group; 2017. p. 73. doi: 10.1136/ejhpharm-2017-001213 31156907 PMC6451557

[34] Yu T, Enkh-Amgalan N, Zorigt G. Methods to perform systematic reviews of patient preferences: A literature survey. BMC Medical Research Methodology. 2017;17. doi: 10.1186/s12874-017-0448-8 29228914 PMC5725984

[35] Tervonen T, Pignatti F, Postmus D. From Individual to Population Preferences: Comparison of Discrete Choice and Dirichlet Models for Treatment Benefit-Risk Tradeoffs. Medical Decision Making. 2019;39: 879–885. doi: 10.1177/0272989X19873630 31496357 PMC6843605

[36] Baltussen R, Mikkelsen E, Tromp N, Hurtig AK, Byskov J, Olsen Ø, et al. Balancing efficiency, equity and feasibility of HIV treatment in South Africa—development of programmatic guidance. Cost Effectiveness and Resource Allocation. 2013;11. doi: 10.1186/1478-7547-11-26 24107435 PMC3851565

[37] Abe JM, Nakamatsu K, Akama S. Two applications of paraconsistent logical controller. Studies in Computational Intelligence. 2008;142: 249–254. doi: 10.1007/978-3-540-68127-4_26

[38] Abe JM, Akama S, Nakamatsu K. Introduction to Annotated Logics. Cham: Springer International Publishing; 2015. doi: 10.1007/978-3-319-17912-4

[39] Elliott Mendelson. Introduction to mathematical logic. New York: Chapman & Hall; 2001.

[40] de Carvalho FR. Paraconsistent logic in decision making: Paraconsistent decision method (PDM). In: Abe J, editor. Paraconsistent Intelligent Based-Systems: New Trends in the Applications of Paraconsistency. Heidelberg: Springer Science and Business Media Deutschland GmbH; 2015. pp. 233–272. doi: 10.1007/978-3-319-19722-7_10

[41] da Costa NCA, Subrahmanian VS, Vago C. The Paraconsistent Logics PJ. Zeitschrift für Mathematische Logik und Grundlagen der Mathematik. 1991;37: 139–148. doi: 10.1002/malq.19910370903

[42] Brasil. Ministério da Saúde. Secretaria de Ciência T e IE. Cetuximabe para o tratamento do câncer colorretal metastático RAS selvagem com doença limitada ao fígado em primeira linha. 2018. Available: http://conitec.gov.br/images/Relatorios/2018/Relatorio_Cetuximabe_CAColorretal_Metastatico.pdf

[43] Hoff P, Diz M, Testa L. Manual de condutas em oncologia. 3.ed. Rio de Janeiro: Atheneu; 2019.

[44] Nebuloni DR, Mak MP, Souza FH, Saragiotto DF, Júlio T, de Castro G, et al. Modified FLOX as first-line chemotherapy for metastatic colorectal cancer patients in the public health system in Brazil: Effectiveness and cost-utility analysis. Molecular and Clinical Oncology. 2013;1: 175–179. doi: 10.3892/mco.2012.12 24649143 PMC3956233

[45] Tournigand C, André T, Achille E, Lledo G, Flesh M, Mery-Mignard D, et al. FOLFIRI followed by FOLFOX6 or the reverse sequence in advanced colorectal cancer: A randomized GERCOR study. Journal of Clinical Oncology. 2004;22: 229–237. doi: 10.1200/JCO.2004.05.113 14657227

[46] Saltz LB, Cox J v., Blanke C, Rosen LS, Fehrenbacher L, Moore MJ, et al. Irinotecan plus fluorouracil and leucovorin for metastatic colorectal cancer. New England Journal of Medicine. 2000;343: 905–914. doi: 10.1056/NEJM200009283431302 11006366

[47] van Cutsem E, Köhne CH, Hitre E, Zaluski J, Chien CRC, Makhson A, et al. Cetuximab and chemotherapy as initial treatment for metastatic colorectal cancer. New England Journal of Medicine. 2009;360: 1408–1417. doi: 10.1056/NEJMoa0805019 19339720

[48] Douillard JY, Siena S, Cassidy J, Tabernero J, Burkes R, Barugel M, et al. Randomized, Phase III trial of panitumumab with infusional fluorouracil, leucovorin, and oxaliplatin (FOLFOX4) Versus FOLFOX4 alone as first-line treatment in patients with previously untreated metastatic colorectal cancer: The PRIME study. Journal of Clinical Oncology. 2010;28: 4697–4705. doi: 10.1200/JCO.2009.27.4860 20921465

[49] Carvalho AC, Leal F, Sasse AD. Cost-effectiveness of cetuximab and panitumumab for chemotherapy-refractory metastatic colorectal cancer. PLoS ONE. 2017;12. doi: 10.1371/journal.pone.0175409 28403233 PMC5389795

[50] Phillips LD, Phillips MC. Facilitated work groups: theory and practice. Journal of the Operational Research Society. 1993;44: 533–549. doi: 10.1057/jors.1993.96

[51] Campolina AG, Suzumura EA, Hong QN, de Soárez PC. Multicriteria decision analysis in health care decision in oncology: a systematic review. Expert Review of Pharmacoeconomics & Outcomes Research. 2021; 1–16. doi: 10.1080/14737167.2022.2019580 34913775

[52] NICE. Bevacizumab and cetuximab for the treatment of metastatic colorectal cancer. Technology Appraisal Guidance 118: National Institute for Health and Care Excellence; 2007. Available: https://www.nice.org.uk/Guidance/TA118

[53] NICE. Bevacizumab in combination with oxaliplatin and either fluorouracil plus folinic acid or capecitabine for the treatment of metastatic colorectal cancer. Technology Appraisal Guidance 212: National Institute for Health and Care Excellence; 2010. Available: https://www.nice.org.uk/guidance/ta212

[54] NICE. Cetuximab, bevacizumab and panitumumab for the treatment of metastatic colorectal cancer after first-line chemotherapy: Cetuximab (monotherapy or combination chemotherapy), bevacizumab (in combination with non-oxaliplatin chemotherapy) and panitumumab (monotherapy) for the treatment of metastatic colorectal cancer after first-line chemotherapy. Technology Appraisal Guidance 242: National Institute for Health and Care Excellence; 2012. Available: https://www.nice.org.uk/guidance/ta242

[55] Kyu HH, Abate D, Abate KH, Abay SM, Abbafati C, Abbasi N, et al. Global, regional, and national disability-adjusted life-years (DALYs) for 359 diseases and injuries and healthy life expectancy (HALE) for 195 countries and territories, 1990–2017: A systematic analysis for the Global Burden of Disease Study 2017. The Lancet. 2018;392: 1859–1922. doi: 10.1016/S0140-6736(18)32335-3 30415748 PMC6252083

[56] Carvalho FR de Abe JM. A Paraconsistent Decision-Making Method. Cham: Springer International Publishing; 2018. doi: 10.1007/978-3-319-74110-9

[57] Angelis A, Montibeller G, Hochhauser D, Kanavos P. Multiple criteria decision analysis in the context of health technology assessment: a simulation exercise on metastatic colorectal cancer with multiple stakeholders in the English setting. doi: 10.1186/s12911-017-0524-3 29073892 PMC5658981

[58] Keeney RL, Raiffa H. Decisions with multiple objectives: preferences and value tradeoffs. Cambridge: Cambridge University Press; 1993.

[59] Drake JI, de Hart JCT, Monleón C, Toro W, Valentim J. Utilization of multiple-criteria decision analysis (MCDA) to support healthcare decision-making FIFARMA, 2016. Journal of Market Access & Health Policy. 2017;5: 1360545. doi: 10.1080/20016689.2017.1360545 29081919 PMC5645903

[60] von Winterfeldt D, Edwards W. Decision analysis and behavioral research. Cambridge: Cambridge University Press; 1986. doi: 10.1111/j.1539-6924.1986.tb00954.x

[61] Fasolo B, Bana e Costa CA. Tailoring value elicitation to decision makers’ numeracy and fluency: Expressing value judgments in numbers or words. Omega (United Kingdom). 2014;44: 83–90. doi: 10.1016/J.OMEGA.2013.09.006

[62] Broekhuizen H, Groothuis-Oudshoorn CGM, van Til JA, Hummel JM, IJzerman MJ. A review and classification of approaches for dealing with uncertainty in multi-criteria decision analysis for healthcare decisions. PharmacoEconomics. Springer International Publishing; 2015. pp. 445–455. doi: 10.1007/s40273-014-0251-x 25630758 PMC4544539

[63] Montibeller G, von Winterfeldt D. Cognitive and Motivational Biases in Decision and Risk Analysis. Risk Analysis. 2015;35: 1230–1251. doi: 10.1111/risa.12360 25873355

