# Supplementary material for: Multiple Criteria Decision Analysis (MCDA) for evaluating cancer treatments in hospital-based health technology assessment: The Paraconsistent Value Framework
Source: PLoS One. 2022 May 25;17(5):e0268584. doi: 10.1371/journal.pone.0268584 (PMC9132343; doi:10.1371/journal.pone.0268584)
Supplement: S1 Table — (DOCX) [file pone.0268584.s001.docx]

**S1 Table. Search strategy for MCDA studies in oncology**

| **Database** | **Search Strategy** | **# references retrieved** |
| --- | --- | --- |
| MEDLINE PubMed | (MCDA OR multi-criteria decision analysis OR multi-criteria decision analyses OR multiple criteria decision aiding OR multi-criteria decision making OR multi-criteria analysis OR multi-attribute utility OR multiple objective problems OR multiattribute decision analysis OR “multi-criteria-decision-analysis” OR “multi-criteria decision analysis” OR “multi criteria decision analysis” OR multiple criteria decision analysis OR “multiple-criteria decision analysis” OR “multiple-criteria-decision analysis” OR “multi-criteria analysis” OR “multiple criteria analysis” OR “multiple-criteria analysis” OR “multiple criteria-analysis” OR “multi-criteria decision making” OR “multi-criteria-decision-making” OR “multi criteria decision making” OR “multiple-criteria decision making” OR “multiple-criteria-decision-making” OR multiple criteria decision making) AND (“Neoplasms” [Mesh] OR oncology OR Cancer OR oncologic patients)  (MCDA OR multi-criteria decision analysis OR multiple criteria decision aiding OR multi-criteria decision making OR multi-criteria analysis OR multi-attribute utility OR multiple objective problems OR multiattribute decision analysis OR “multi-criteria-decision-analysis” OR “multi-criteria decision analysis” OR “multi criteria decision analysis” OR multiple criteria decision analysis OR “multiple-criteria decision analysis” OR “multiple-criteria-decision analysis” OR “multi-criteria analysis” OR “multiple criteria analysis” OR “multiple-criteria analysis” OR “multiple criteria-analysis” OR “multi-criteria decision making” OR “multi-criteria-decision-making” OR “multi criteria decision making” OR “multiple-criteria decision making” OR “multiple-criteria-decision-making” OR multiple criteria decision making) AND (“Neoplasms” [Mesh] OR oncology OR Cancer OR oncologic patients) | 941 |
| EMBASE | (MCDA or 'multi-criteria decision analysis'/exp OR 'multi-criteria decision analysis' OR (multi-criteria AND decision AND analyses) OR (multiple AND criteria AND decision AND aiding) OR 'multi-criteria decision making'/exp OR 'multi-criteria decision making' OR (multi-criteria AND analysis) OR (multi-attribute AND utility) OR (multiple AND objective AND problems) OR 'multiple criteria decision analysis'/exp OR 'multiple criteria decision analysis' OR (multi-criteria AND ('analysis'/exp OR analysis)) OR ('multi criteria' AND ('decision'/exp OR decision) AND making) OR ('multiple criteria' AND ('decision'/exp OR decision) AND making)) AND ('malignant neoplasm'/exp OR 'malignant neoplasm' OR 'cancer'/exp OR Cancer OR 'oncology'/exp OR oncology OR (oncologic AND patients)) | 790 |
| Web of Science | TS=(MCDA OR “multi-criteria decision analysis” OR “multi-criteria decision analyses” OR “multiple criteria decision aiding” OR “multi-criteria decision making” OR “multi-criteria analysis” OR “multi-attribute utility” OR “multiple objective problems” OR “multiple criteria decision analysis” OR “multiple criteria decision making” OR “multi-criteria analyses” OR “multiattribute decision analysis” OR “multi-criteria-decision-analysis” OR “multi-criteria decision analysis” OR “multi criteria decision analysis” OR “multiple criteria decision analysis” OR “multiple-criteria decision analysis” OR “multiple-criteria-decision analysis” OR “multi-criteria analysis” OR “multi-criteria analysis” OR “multiple criteria analysis” OR “multiple-criteria analysis” OR “multiple criteria-analysis” OR “multi-criteria decision making” OR “multi-criteria-decision-making” OR “multi criteria decision making” OR “multiple-criteria decision making” OR “multiple-criteria-decision-making” OR “multiple criteria decision making”) AND TS=(“malignant neoplasm” OR Neoplasms OR cancer OR oncology OR “oncologic patients”)  Índices=SCI-EXPANDED, SSCI, A&HCI, CPCI-S, CPCI-SSH, ESCI Tempo estipulado=Todos os anos | 121 |
| LILACS | (tw:((tw:(MCDA OR “multi-criteria decision analysis” OR “multi-criteria decision analyses” OR “multiple criteria decision aiding” OR “multi-criteria decision making” OR “multi-criteria analysis” OR “multi-attribute utility” OR “multiple objective problems” OR “multiattribute decision analysis” OR “multi-criteria-decision-analysis” OR “multi-criteria decision analysis” OR “multi criteria decision analysis” OR multiple criteria decision analysis OR “multiple-criteria decision analysis” OR “multiple-criteria-decision analysis” OR “multi-criteria analysis” OR “multiple criteria analysis” OR “multiple-criteria analysis” OR “multiple criteria-analysis” OR “multi-criteria decision making” OR “multi-criteria-decision-making” OR “multi criteria decision making” OR “multiple-criteria decision making” OR “multiple-criteria-decision-making” OR “multiple criteria decision making”)) OR (tw:( “Técnicas de Apoio para a Decisão” OR “Decision Support Techniques” OR “Técnicas de Apoyo para la Decisión” OR “Análise de decisão multicritério”)))) AND (tw:(Neoplasms OR Neoplasias OR oncology OR Cancer OR Cancro OR Neoplasmas OR Tumor OR Tumores OR Neoplasia)) | 10 |
| CRD NHS EED | (MCDA OR multi-criteria decision analysis OR multi-criteria decision analyses OR multiple criteria decision aiding OR multi-criteria decision making OR multi-criteria analysis OR multi-attribute utility OR multiple objective problems OR multiple criteria decision analysis OR multiple criteria decision making OR multi-criteria analyses OR multiattribute decision analysis) IN NHSEED | 9 |
| **TOTAL** |  | **1871** |

Date: 10/Nov/2018
