# Supplementary material for: Multiple Criteria Decision Analysis (MCDA) for evaluating cancer treatments in hospital-based health technology assessment: The Paraconsistent Value Framework
Source: PLoS One. 2022 May 25;17(5):e0268584. doi: 10.1371/journal.pone.0268584 (PMC9132343; doi:10.1371/journal.pone.0268584)
Supplement: S2 Table — (DOCX) [file pone.0268584.s002.docx]

**S2 Table.** **Performance scores (bivalued annotations) assigned by experts**

| Oncology | | Surgery | | Radiotherapy | | Palliative | | Management | | Economics | | Epidemiology | | Pharmacy | | Industry | | Media | | Patient | |
| --- | --- | --- | --- | --- | --- | --- | --- | --- | --- | --- | --- | --- | --- | --- | --- | --- | --- | --- | --- | --- | --- |
| *a*_i,j,1_ | ***b***_i,j,1_ | ***a***_i,j,2_ | ***b***_i,j,2_ | ***a***_i,j,3_ | ***b***_i,j,3_ | ***a***_i,j,4_ | ***b***_i,j,4_ | ***a***_i,j,5_ | ***b***_i,j,5_ | ***a***_i,j,6_ | ***b***_i,j,6_ | ***a***_i,j,7_ | ***b***_i,j,7_ | ***a***_i,j,8_ | ***b***_i,j,8_ | ***a***_i,j,9_ | ***b***_i,j,9_ | ***a***_i,j,10_ | ***b***_i,j,10_ | ***a***_i,j,11_ | ***b***_i,j,11_ |
| 1.00 | 0.10 | 0.90 | 0.20 | 0.90 | 0.10 | 0.85 | 0.05 | 0.80 | 0.30 | 1.00 | 0.00 | 0.90 | 0.15 | 0.94 | 0.02 | 0.78 | 0.45 | 0.97 | 0.12 | 0.95 | 0.00 |
| 1.00 | 0.15 | 0.80 | 0.20 | 0.75 | 0.10 | 0.85 | 0.13 | 0.70 | 0.30 | 1.00 | 0.00 | 0.80 | 0.25 | 0.90 | 0.07 | 0.78 | 0.45 | 0.70 | 0.12 | 0.90 | 0.00 |
| 1.00 | 0.15 | 0.80 | 0.20 | 0.70 | 0.10 | 0.75 | 0.05 | 0.62 | 0.30 | 1.00 | 0.00 | 0.70 | 0.35 | 0.75 | 0.28 | 0.78 | 0.45 | 0.50 | 0.30 | 0.90 | 0.00 |
| 1.00 | 0.15 | 0.70 | 0.30 | 0.65 | 0.05 | 0.70 | 0.05 | 0.58 | 0.30 | 0.90 | 0.00 | 0.60 | 0.40 | 0.66 | 0.50 | 0.78 | 0.45 | 0.35 | 0.50 | 0.90 | 0.00 |
| 0.50 | 0.95 | 0.30 | 0.10 | 0.60 | 0.05 | 0.50 | 0.20 | 0.50 | 0.40 | 0.70 | 0.70 | 0.20 | 0.43 | 0.40 | 0.02 | 0.70 | 0.45 | 0.18 | 0.65 | 0.60 | 0.50 |
| 0.85 | 0.50 | 0.60 | 0.30 | 0.85 | 0.20 | 0.90 | 0.05 | 0.80 | 0.30 | 1.00 | 0.00 | 0.90 | 0.20 | 0.90 | 0.40 | 0.65 | 0.55 | 0.98 | 0.02 | 0.80 | 0.20 |
| 0.85 | 0.50 | 0.60 | 0.30 | 0.60 | 0.18 | 0.80 | 0.05 | 0.75 | 0.30 | 0.90 | 0.00 | 0.85 | 0.23 | 0.80 | 0.40 | 0.65 | 0.55 | 0.70 | 0.20 | 0.68 | 0.20 |
| 0.50 | 0.70 | 0.40 | 0.40 | 0.50 | 0.10 | 0.80 | 0.20 | 0.69 | 0.30 | 0.80 | 0.10 | 0.74 | 0.30 | 0.68 | 0.20 | 0.65 | 0.55 | 0.50 | 0.32 | 0.68 | 0.20 |
| 0.50 | 0.70 | 0.30 | 0.40 | 0.40 | 0.10 | 0.70 | 0.20 | 0.66 | 0.35 | 0.70 | 0.29 | 0.30 | 0.15 | 0.55 | 0.20 | 0.65 | 0.55 | 0.34 | 0.65 | 0.56 | 0.10 |
| 0.20 | 0.90 | 0.15 | 0.15 | 0.20 | 0.05 | 0.40 | 0.20 | 0.55 | 0.50 | 0.50 | 0.40 | 0.20 | 0.08 | 0.45 | 0.09 | 0.55 | 0.55 | 0.27 | 0.65 | 0.30 | 0.00 |
| 0.95 | 0.05 | 0.90 | 0.10 | 0.99 | 0.00 | 0.90 | 0.05 | 0.90 | 0.20 | 1.00 | 0.00 | 0.80 | 0.01 | 0.99 | 0.00 | 0.30 | 0.37 | 0.80 | 0.20 | 0.90 | 0.10 |
| 0.75 | 0.40 | 0.80 | 0.20 | 0.97 | 0.00 | 0.80 | 0.05 | 0.80 | 0.27 | 0.70 | 0.10 | 0.68 | 0.06 | 0.84 | 0.05 | 0.22 | 0.32 | 0.50 | 0.30 | 0.70 | 0.00 |
| 0.50 | 0.30 | 0.70 | 0.20 | 0.50 | 0.25 | 0.50 | 0.15 | 0.70 | 0.30 | 0.50 | 0.20 | 0.60 | 0.08 | 0.45 | 0.08 | 0.13 | 0.27 | 0.35 | 0.30 | 0.56 | 0.00 |
| 0.30 | 0.30 | 0.40 | 0.10 | 0.03 | 0.00 | 0.20 | 0.15 | 0.55 | 0.38 | 0.30 | 0.20 | 0.35 | 0.08 | 0.20 | 0.03 | 0.04 | 0.20 | 0.18 | 0.20 | 0.41 | 0.00 |
| 0.15 | 0.10 | 0.20 | 0.10 | 0.01 | 0.00 | 0.10 | 0.05 | 0.40 | 0.50 | 0.00 | 0.20 | 0.05 | 0.03 | 0.02 | 0.06 | 0.01 | 0.20 | 0.01 | 0.30 | 0.30 | 0.00 |
| 0.90 | 0.60 | 0.90 | 0.07 | 1.00 | 0.00 | 0.95 | 0.02 | 0.85 | 0.20 | 1.00 | 0.00 | 0.92 | 0.05 | 0.91 | 0.01 | 0.70 | 0.50 | 0.98 | 0.00 | 0.85 | 0.10 |
| 0.85 | 0.60 | 0.90 | 0.12 | 0.98 | 0.00 | 0.95 | 0.10 | 0.78 | 0.25 | 1.00 | 0.00 | 0.87 | 0.10 | 0.80 | 0.03 | 0.60 | 0.50 | 0.88 | 0.00 | 0.85 | 0.20 |
| 0.85 | 0.60 | 0.80 | 0.12 | 0.93 | 0.02 | 0.80 | 0.10 | 0.65 | 0.30 | 1.00 | 0.00 | 0.82 | 0.18 | 0.73 | 0.03 | 0.55 | 0.55 | 0.88 | 0.00 | 0.70 | 0.20 |
| 0.70 | 0.50 | 0.80 | 0.21 | 0.80 | 0.15 | 0.70 | 0.10 | 0.55 | 0.45 | 1.00 | 0.00 | 0.75 | 0.22 | 0.68 | 0.05 | 0.51 | 0.55 | 0.88 | 0.00 | 0.70 | 0.35 |
| 0.50 | 0.15 | 0.65 | 0.21 | 0.60 | 0.30 | 0.55 | 0.02 | 0.43 | 0.55 | 0.85 | 0.15 | 0.66 | 0.30 | 0.52 | 0.06 | 0.51 | 0.60 | 0.60 | 0.99 | 0.35 | 0.00 |
| 0.90 | 0.05 | 0.50 | 0.40 | 0.90 | 0.10 | 0.90 | 0.05 | 0.80 | 0.30 | 0.80 | 0.60 | 0.85 | 0.20 | 0.99 | 0.02 | 0.85 | 0.26 | 0.95 | 0.04 | 0.90 | 0.30 |
| 0.90 | 0.15 | 0.40 | 0.40 | 0.80 | 0.20 | 0.80 | 0.05 | 0.70 | 0.30 | 0.60 | 0.70 | 0.78 | 0.13 | 0.97 | 0.10 | 0.78 | 0.26 | 0.90 | 0.04 | 0.80 | 0.30 |
| 0.80 | 0.25 | 0.40 | 0.50 | 0.70 | 0.20 | 0.70 | 0.16 | 0.60 | 0.30 | 0.40 | 0.80 | 0.69 | 0.30 | 0.76 | 0.30 | 0.73 | 0.32 | 0.78 | 0.15 | 0.80 | 0.15 |
| 0.70 | 0.60 | 0.27 | 0.50 | 0.70 | 0.20 | 0.65 | 0.25 | 0.50 | 0.30 | 0.30 | 0.95 | 0.65 | 0.39 | 0.61 | 0.33 | 0.73 | 0.36 | 0.61 | 0.35 | 0.65 | 0.00 |
| 0.10 | 0.95 | 0.27 | 0.50 | 0.50 | 0.15 | 0.50 | 0.25 | 0.47 | 0.40 | 0.30 | 0.95 | 0.54 | 0.48 | 0.21 | 0.17 | 0.66 | 0.36 | 0.45 | 0.45 | 0.50 | 0.00 |
