# Supplementary material for: Multiple Criteria Decision Analysis (MCDA) for evaluating cancer treatments in hospital-based health technology assessment: The Paraconsistent Value Framework
Source: PLoS One. 2022 May 25;17(5):e0268584. doi: 10.1371/journal.pone.0268584 (PMC9132343; doi:10.1371/journal.pone.0268584)
Supplement: S3 Table — (DOCX) [file pone.0268584.s003.docx]

**S3 Table. Criteria weights assigned by experts**

|  | OS | PFS | AEs | CT | BoD |
| --- | --- | --- | --- | --- | --- |
| Oncology | 0.2857 | 0.1000 | 0.2286 | 0.2143 | 0.1714 |
| Surgery | 0.2857 | 0.1143 | 0.2000 | 0.1714 | 0.2286 |
| Radiotherapy | 0.2299 | 0.2069 | 0.2184 | 0.1609 | 0.1839 |
| Palliative | 0.1609 | 0.1839 | 0.2184 | 0.2069 | 0.2299 |
| Management | 0.1889 | 0.1778 | 0.2111 | 0.2000 | 0.2222 |
| Economics | 0.2093 | 0.2326 | 0.1860 | 0.1977 | 0.1744 |
| Epidemiology | 0.2353 | 0.2941 | 0.2059 | 0.0882 | 0.1765 |
| Pharmacy | 0.2564 | 0.1795 | 0.1282 | 0.2051 | 0.2308 |
| Industry | 0.2128 | 0.1773 | 0.1844 | 0.1891 | 0.2364 |
| Media | 0.2857 | 0.1000 | 0.2286 | 0.2143 | 0.1714 |
| Patient | 0.2857 | 0.1143 | 0.2000 | 0.1714 | 0.2286 |

OS=overall survival; PFS=progression free survival; AEs=grade 3 and 4 adverse events; CT=cost of treatment; BoD=burden of disease.
