# Supplementary material for: Multiple Criteria Decision Analysis (MCDA) for evaluating cancer treatments in hospital-based health technology assessment: The Paraconsistent Value Framework
Source: PLoS One. 2022 May 25;17(5):e0268584. doi: 10.1371/journal.pone.0268584 (PMC9132343; doi:10.1371/journal.pone.0268584)
Supplement: S1 Fig — (DOCX) [file pone.0268584.s004.docx]

**S1 Fig. One-way sensitivity analysis on criteria weights for first line metastatic colorectal cancer**

E1= expert 1 (medical oncology); E2 = expert 2 (oncologic surgery); E3 = expert 3 (radiotherapy); E4 = expert 4 (palliative care); E5 = expert 5 (pharmacist); E6 = expert 6 (health economist); E7 = expert 7 (epidemiologist); E8 = expert 8 (public health expert); E9 = expert 9 (health media expert), E10 = expert 10 (pharmaceutical industry); E11 = expert 11 (patient advocate)
