# Supplementary material for: Multiple Criteria Decision Analysis (MCDA) for evaluating cancer treatments in hospital-based health technology assessment: The Paraconsistent Value Framework
Source: PLoS One. 2022 May 25;17(5):e0268584. doi: 10.1371/journal.pone.0268584 (PMC9132343; doi:10.1371/journal.pone.0268584)
Supplement: S2 Fig — (DOCX) [file pone.0268584.s005.docx]

**S2 Fig. One-way sensitivity analysis on control levels for first-line metastatic colorectal cancer**

1= modified Flox (5FU, oxaliplatin, leucovorin); 2= modified IFL (irinotecan, 5FU, leucovorin); 3= modified FOLFOX6 (5FU, oxaliplatin, leucovorin); 4= FOLFIRI (5FU, oxaliplatin, irinotecan, leucovorin); 5=Panitumumab; 6=Cetuximab.

1= modified Flox (5FU, oxaliplatin, leucovorin); 2= modified IFL (irinotecan, 5FU, leucovorin); 3= modified FOLFOX6 (5FU, oxaliplatin, leucovorin); 4= FOLFIRI (5FU, oxaliplatin, irinotecan, leucovorin); 5=Panitumumab; 6=Cetuximab.

1= modified Flox (5FU, oxaliplatin, leucovorin); 2= modified IFL (irinotecan, 5FU, leucovorin); 3= modified FOLFOX6 (5FU, oxaliplatin, leucovorin); 4= FOLFIRI (5FU, oxaliplatin, irinotecan, leucovorin); 5=Panitumumab; 6=Cetuximab.
