# Supplementary material for: Multiple Criteria Decision Analysis (MCDA) for evaluating cancer treatments in hospital-based health technology assessment: The Paraconsistent Value Framework
Source: PLoS One. 2022 May 25;17(5):e0268584. doi: 10.1371/journal.pone.0268584 (PMC9132343; doi:10.1371/journal.pone.0268584)
Supplement: S1 File — (DOCX) [file pone.0268584.s006.docx]

**S1 File. Primer on Annotated Evidential Logic**

**Introduction**

Intuitively, in Annotated Evidential Logic (Logic Eτ), what is done is to assign an annotation (μ1; μ2), with μ1 and μ2 belonging to the closed interval [0; 1], for each elementary (atomic) proposition p in such a way that μ1 translates the degree of belief (or favorable evidence) that is found in p and μ2, the degree of disbelief (or contrary evidence).

The set [0; 1]^2^ or [0; 1] X [0; 1] endowed with an order relationship * such that (λ1; λ2) * (μ1; μ2) if and only if λ1 ≤ μ1 and λ2 ≤ μ2, where ≤ is the usual total order ratio of real numbers, constitutes a lattice of annotations (Figure 1). Each pair (μ1; μ2) constitutes a logical state. The following extreme logical states stand out:

(1; 0) represents, intuitively, total belief and no disbelief (translates a logical state called truth, which is represented by V);

(0; 1) intuitively represents no belief and total disbelief (translates a logical state called falsehood, which is represented by F);

(1; 1) intuitively represents total belief and disbelief (translates a logical state called inconsistency, which is represented by ┬), and

(0; 0) indicates total absence of belief and disbelief (translates a logical state called para-completeness or indeterminacy, which is represented by ⊥).

The annotation grid can be divided in several ways. A convenient division is that which structures the lattice in twelve regions, as in Figure 1, where the result of the analyzes is synthesized, based on degrees of certainty and contradiction. In Figure 1, four extreme regions and a central region can be highlighted:

AMN region: -1 ≤ Gcontr ≤ - 0.60 ⇒ para-completion region.

BRS region: 0.60 ≤ Gcontr ≤ 1 ⇒ region of inconsistency.

CPQ region: 0.60 ≤ H cert ≤ 1 ⇒ true region.

DTU region: -1 ≤ H cert ≤ - 0.60 ⇒ falsehood region.

Region MNTUSRQP: | G contr | <0.60 or - 0.60 <G contr <0.60 and | H cert | <0.60 or - 0.60 <H cert <0.60.

Among the extreme regions, the CPQ and DTU regions are called decision regions. The first, with a favorable decision (truth) and the second, with an unfavorable decision (falsehood), as described in Chart 1.

The central MNTUSRQP region (with its sub-regions) does not allow definitive conclusions, that is, when the point that translates the analysis result belongs to that region, we say that the analysis expresses trends, designated by the term “almost”, as described in Table 1.

Figure 1 - Decision rule and para-analyzer algorithm for a level of requirement equal to 0.70

AB = Perfectly Undefined Line (PIL); CD = Perfectly defined line (PDL); AM = Border line of para-completeness; AMN = Region of para-completeness; RS = Border line of inconsistency BRS = Region of Inconsistency; PQ = Border line of truth; CPQ = Region of truth (or favorable decision); TU = Border line of falsity; DTU = Region of falsity (or unfavorable decision)

Table 1 - Summary of the analysis of the twelve regions of Cartesian Unitary Square

| Region | a | b | G | H | Description | Representation |
| --- | --- | --- | --- | --- | --- | --- |
| AMN | [0; 0.4] | [0; 0.4] | [−1; −0.6] | [−0.4; 0.4] | Indetermination or  paracompleteness | ⊥ |
| BRS | [0.6; 1] | [0.6; 1] | [0.6; 1] | [−0.4; 0.4] | Inconsistency | ┬ |
| CPQ | [0.6; 1] | [0; 0.4] | [−0.4; 0.4] | [0.6; 1] | Truth | V |
| DTU | [0; 0.4] | [0.6; 1] | [−0.4; 0.4] | [−1; −0.6] | Falsity | F |
| OFSL | [0.5; 0.8] | [0.5; 1] | [0; 0.6[ | [−0.5; 0] | Quasi-inconsistency  tending to falsity | Q┬ → F |
| OHUL | [0.2; 0.5] | [0.5; 1] | [0; 0.5] | [−0.6; 0] | Quasi-falsity tending  to inconsistency | QF → ┬ |
| OHTI | [0; 0.5] | [0.5; 0.8] | [–0.5; 0] | [−0.6; 0] | Quasi-falsity tending  to indetermination | QF → ┴ |
| OENI | [0; 0.5] | [0.2; 0.5] | [−0.6; 0] | [−0.5; 0] | Quasi-indetermination  tending to falsity | Q┴ → F |
| OEMK | [0.2; 0.5] | [0; 0.5] | [−0.6; 0] | [0; 0.5] | Quasi-indetermination  tending to truth | Q┴ → V |
| OGPK | [0.5; 0.8] | [0; 0.5] | [−0.5; 0] | [0; 0.6] | Quasi-truth tending to  indetermination | QV → ┴ |
| OGQJ | [0.5; 1] | [0.2; 0.5] | [0; 0.5] | [0; 0.6] | Quasi-truth tending to  inconsistency | QV → ┬ |
| OFRJ | [0.5; 1] | [0.5; 0.8] | [0; 0.6] | [0; 0.5] | Quasi-inconsistency  tending to truth | Q┬ → V |

**Settings**

Degree of contradiction: G contr = μ1 + μ2 - 1, where you can conclude: -1 ≤ G contr ≤ 1.

Degree of certainty: H cert = μ1 - μ2, where you can conclude: -1 ≤ H cert ≤ 1.

Para-completion limit line: MN segment, which G contr = - k1, for 0 <k1 <1.

Inconsistency limit line: segment RS, where G contr = + k1, for 0 <k1 <1.

False limit line: segment TU, which H cert = - k2, for 0 <k2 <1.

Truth limit line: PQ segment, which H cert = + k2, for 0 <k2 <1.

**Steps of the Paraconsistent Decision Method**

The Paraconsistent Decision Method (PDM) consists of eight steps, of which only a brief idea will be outlined at first, while the rest of the details will come along shortly down the chapter.

(1) Set the level of the requirement (LR) or control level of the decision to be made.

(2) Select the most critical factors (Fi) that most influence the decision.

(3) Define sections (Sj) for each factor (Three, four, five, or more sections can be set depending on the case and the level of detail desired).

(4) Build the database, which is composed of the weights (Pi) assigned to factors (for instance to distinguish them by importance) and by the values of favorable evidence (or degree of belief) (a) and the contrary evidence (or degree of disbelief) (b) assigned to each factor in one of the sections; the weights and values of evidence are assigned by experts conveniently selected to give their opinion (The database can also be built with stored statistical data obtained from previous experiences in similar enterprises).

(5) Perform field survey (or research) to find out in which section (condition) each factor is placed.

(6) Obtain the value of the degree of favorable evidence (ai,R) and the value of the degree of contrary evidence (bi,R) for each of the chosen factors (Fi), with 1 ≤ i ≤ n, in sections found in the survey (Spj) by applying the maximizing (MAX operator) and minimizing (MIN operator) techniques of Logic (Eτ).

(7) Obtain the degree of favorable evidence (aW) and the degree of contrary evidence (bW) of the barycenter of the points representing the selected factors in the lattice (τ).

(8) Make the decision by applying the decision rule or the para-analyzing algorithm.
